# Coumarin Probe for Selective Detection of Fluoride Ions in Aqueous Solution and Its Bioimaging in Live Cells

**DOI:** 10.3390/s18072042

**Published:** 2018-06-26

**Authors:** Kantapat Chansaenpak, Anyanee Kamkaew, Oratai Weeranantanapan, Khomson Suttisintong, Gamolwan Tumcharern

**Affiliations:** 1 National Nanotechnology Center, National Science and Technology Development Agency, Thailand Science Park, Pathum Thani 12120, Thailand; khomson@nanotec.or.th (K.S.); gamolwan@nanotec.or.th (G.T.); 2 School of Chemistry, Institute of Science, Suranaree University of Technology, Nakhon Ratchasima 30000, Thailand; anyanee@sut.ac.th; 3 School of Preclinical Sciences, Institute of Science, Suranaree University of Technology, Nakhon Ratchasima 30000, Thailand; oratai@g.sut.ac.th

**Keywords:** coumarin, chemosensor, fluoride, bioimaging, fluorescence

## Abstract

We have synthesized novel coumarin-based fluorescent chemosensors for detection of fluoride ions in aqueous solution. The detection mechanism relied on a fluoride-mediated desilylation triggering fluorogenic reaction and a strong interaction between fluoride and the silicon center. In this work, the hydroxyl-decorated coumarins containing oxysilyl moiety have been synthesized through the aldehyde-functionalized coumarins. The optical responses toward fluoride, as well as aqueous stability studies of both aldehyde and hydroxyl functionalized coumarins, have been investigated. Due to the highest fluorescence enhancement upon the addition of fluoride and good stability in aqueous solution, the hydroxyl-decorated coumarin connected with the bulky *tert*-butyldiphenyloxysilyl group (-OSi*t*BuPh_2_) has been selected for further investigation of its potential as a fluoride sensor. This hydroxyl-decorated coumarin can selectively sense fluoride ions in aqueous media (contain 0.8% MeCN) with desirable response times (40 min). The limit of detection of this compound was determined as 0.043 ppm, satisfying the standard fluoride level (0.7 ppm) in drinking water recommended by U.S. Department of Health and Human Services. The application of this silyl-capped coumarin derivative for fluoride analysis in collected water samples displayed satisfactory analytical accuracy (<5% error). Finally, this compound was successfully employed in fluorescence bioimaging of fluoride ions in human liver cancer cells, indicating its excellent cell permeability, ability to retain inside the living cells, and good stability under physiological conditions.

## 1. Introduction

The fluoride ion (F^−^) is of special interest owing to its important role in tooth decay prevention [1], osteoporosis treatment [2], and medical diagnosis by positron emission tomography (PET) [3,4,5,6]. Nevertheless, overexposure to fluoride can lead to adverse health effects, such as dental/skeletal fluorosis, and acute gastric and kidney problems, because fluoride is readily absorbed by the human body, but is excreted slowly [7,8,9]. Recently, high fluoride levels in drinking water have been linked to neurodevelopmental disabilities in children [10]. These detrimental health effects have sparked growing attention in the development of fluoride sensors at part-per-million (ppm) levels in aqueous phase and biological systems.

Owing to their high sensitivities, low detection limits, and potential to be applied for bioimaging, fluorescence sensor systems have attracted the highest interest [11,12]. However, there are challenges [11] regarding the development of fluorogenic molecular sensors for fluoride detection in water that need to be addressed, namely: (1) the water-solubility of molecular sensors, (2) the instability of fluoride-responsive moiety in water, (3) the poor fluorescence signal of molecular sensors in aqueous media, and (4) the hydration of fluoride anion. One established approach to constructing fluoride-selective fluorescence sensors hinges on the formation of strong interactions between fluoride and a Lewis acidic silicon center through Si-O bond cleavage [13]. This approach has been employed in various fluorescent dyes, such as, naphthalimide [14,15,16,17,18,19], fluorescein [20,21], BODIPY [22,23], benzothiazole [24,25,26], and coumarin [24,25,26]. Although several fluoride-sensing platforms have been demonstrated, only a few examples including dyes decorated with methyl ester [27], ammonium [26], and polyethylene glycol (PEG) [21], can be employed in the purely aqueous phase. These findings indicated that the introduction of hydrophilic moieties into dyes could improve their hydrophilicity, and therefore, enhance their capability to detect fluoride ions in 100% water.

**Figure 1:**
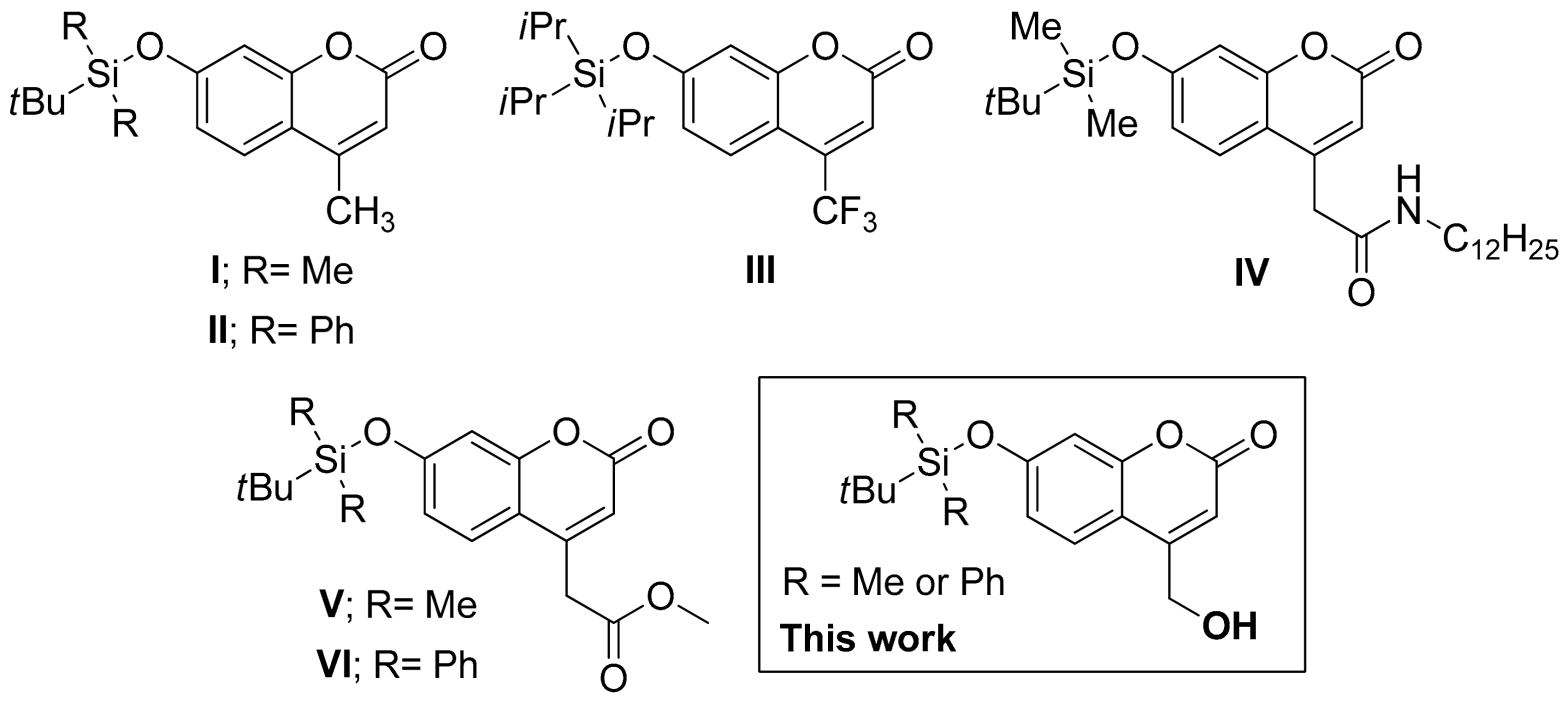
Chemical structures of coumarin-based sensors for fluoride ions reported in previous publications (**I**–**VI**) and in this work.

In the case of coumarin-based sensors (Figure 1), **I** [28] and **II** [29] can detect inorganic fluoride salt (NaF) in an acetone-water solution (7:3 *v*/*v*) and a THF-water solution (4% THF *v*/*v*), respectively. **III** [30] can efficiently detect NaF in an MeCN-water solution (5% MeCN *v*/*v*) in the presence of 18-crown-6 as a cation chelating agent, while **IV** [31] demonstrated sol-to-gel transition upon addition of NaF in a methanol-water solution (1:1 *v*/*v*). Only **VI** [27] can detect fluoride ions (NaF) in completely aqueous media and live cells; however, long incubation times (4 h) with fluoride could be inconvenient in practical applications. In this work, we introduce a hydroxyl moiety to the silyl-capped coumarin backbone (Figure 1). Exploiting the benefits from the hydroxyl group, such as its small size, polarity, and ability to form hydrogen bonds with water, this new hydroxyl-decorated coumarin is expected to demonstrate excellent water solubility, and efficiently detect inorganic fluoride salt in aqueous solutions and live cells with fast response times. Detailed synthesis and fluoride sensing abilities of this compound are presented in this report.

## 2. Materials and Methods

### 2.1. Materials

Compounds **1**, **6**, and MnO_2_ were synthesized according to the published procedures [32,33]. Resorcinol, ethyl 4-chloroacetoacetate, *tert*-butyldiphenylsilyl chloride, *tert*-butyldimethylsilyl chloride, triethylamine, NaOAc, NaBr, and NaI were purchased from TCI Chemicals; NaBH_4_, MnSO_4_, H_2_SO_4_, Na_2_S_2_O_3_·5H_2_O, Na_2_SO_4_, Na_2_CO_3_, NaHCO_3_, NaNO_3_, NaNO_2_, and NaN_3_ were purchased from CARLO ERBA Reagents; KMnO_4_, NaH_2_PO_4_·2H_2_O, and Na_2_HPO_4_ were purchased from Ajax Finechem; NaF and NaCl were purchased from Merck KGaA; Na_3_PO_4_·12H_2_O was purchased from Sigma Aldrich; imidazole, dichloromethane (99.8%, Dried over molecular sieves), and tetrahydrofuran (99.5%, Dried over molecular sieves) were purchased from Acros Organics; hexane and ethyl acetate (ACS grade) were purchased from Honeywell. All chemicals were used without further purification. Electrospray mass spectra were obtained from a Bruker micrOTOF spectrometer. NMR spectra were recorded on a Bruker NMR 500 MHz spectrometer at ambient temperature. Chemical shifts are given in ppm, and are referenced to residual ^1^H and ^13^C solvent signals. UV-VIS absorption and fluorescence spectra were acquired from a Cary Series UV-Vis-NIR spectrophotometer (Agilent Tech, Santa Clara, CA, USA) and a Perkin Elmer LS55 fluorescence spectrometer, respectively. Analytical high-performance liquid chromatography (HPLC) was accomplished on 1260 infinity II LC systems (Agilent Technologies).

### 2.2. Synthesis of 7-((Tert-butyldimethylsilyl)oxy)-2-oxo-2H-chromene-4-carbaldehyde *(**2**)*

To a suspension of **1** (0.1465 g, 0.7704 mmol) in CH_2_Cl_2_ (40 mL) was added imidazole (0.0788 g, 1.1571 mmol) and NEt_3_ (0.22 mL, 1.5408 mmol), and the resulting mixture was stirred for 10 min yielding an orange solution. Then, *tert*-butyldimethylsilyl chloride (0.1740 g, 1.1545 mmol) was added to the orange solution giving white suspension. After being stirred overnight, the reaction mixture was quenched with water (60 mL) and extracted by CH_2_Cl_2_ (3 × 50 mL). The organic layer was separated and washed with brine (3 × 50 mL). The organic layer was dried over anhydrous sodium sulphate, filtered, and the solvent was removed under reduced pressure to give a yellow oil. The crude mixture was purified by column chromatography (Hexane: EtOAc 9:1) yielding yellow powder as a pure product (**2**) (0.1059 g, 45% yield). ^1^H-NMR (500 MHz, CDCl_3_): δ 0.26 (s, 6H, -C*H*_3_), 0.99 (s, 9H, -C*H*_3_), 6.72 (s, 1H, phenyl-C*H*), 6.82–6.86 (m, 2H, phenyl-C*H*), 8.45 (d, 1H, phenyl-C*H*, *J*_H-H_ = 8.6 Hz), 10.07 (s, 1H, -C*H*O). ^13^C-NMR (125 MHz, CDCl_3_): δ 18.50, 25.76, 108.13, 109.05, 118.42, 122.61, 127.58, 144.00, 156.43, 160.35, 160.94, 191.91. HRMS *m*/*z* calculated for C_16_H_20_NaO_4_Si ([M + Na]^+^): 327.1023 found: 327.1013.

### 2.3. Synthesis of 7-((Tert-butyldiphenylsilyl)oxy)-2-oxo-2H-chromene-4-carbaldehyde *(**3**)*

To a suspension of **1** (0.1596 g, 0.8393 mmol) in CH_2_Cl_2_ (40 mL) was added imidazole (0.0857 g, 1.2590 mmol) and NEt_3_ (0.23 mL, 1.6800 mmol). The mixture was stirred for 10 min, yielding an orange solution. Then, *tert*-butyldiphenylsilyl chloride (0.33 mL, 1.2588 mmol) was added to the orange solution giving a yellow solution. After being stirred overnight, the reaction mixture was quenched with water (60 mL) and extracted with dichloromethane (3 × 50 mL). The organic layer was separated and washed with brine (3 × 50 mL). The organic layer was then dried over anhydrous sodium sulphate, filtered, and the solvent was removed under reduced pressure, yielding a yellow oil. The crude mixture was purified by column chromatography (Hexane: EtOAc 4:1) yielding a yellow oil which solidified overnight as a pure product (**3**) (0.2485 g, 69% yield). ^1^H-NMR (500 MHz, CDCl_3_) δ 1.10 (s, 9H, –C*H*_3_), 6.64 (s, 1H, phenyl-C*H*), 6.70 (d, 1H, phenyl-C*H*, *J*_H-H_ = 2.1 Hz), 6.77 (dd, 1H, phenyl-C*H*, *J*_H-H_ = 8.9, 2.1 Hz), 7.37 (dd, 4H, phenyl-C*H*, *J*_H-H_ = 7.3 Hz), 7.44 (t, 2H, phenyl-C*H*, *J*_H-H_ = 7.3 Hz), 7.68 (d, 4H, phenyl-C*H*, *J*_H-H_ = 6.9 Hz), 8.30 (d, 1H, phenyl-C*H*, *J*_H-H_ = 8.9 Hz), 10.00 (s, 1H, -C*H*O). ^13^C-NMR (125 MHz, CDCl_3_) δ 19.43, 26.35, 107.83, 108.69, 117.97, 122.28, 127.08, 128.05, 130.38, 131.52, 135.35, 143.67, 155.59, 159.81, 160.71, 191.64. HRMS *m*/*z* calculated for C_26_H_24_NaO_4_Si ([M + Na]^+^): 451.1336, found: 451.1339.

### 2.4. Synthesis of 7-((Tert-butyldimethylsilyl)oxy)-4-(hydroxyl methyl)-2H-chromen-2-one *(**4**)*

To a solution of **2** (0.5208 g, 1.7109 mmol) in MeOH:THF (1:1) (30 mL) at 0 °C was added NaBH_4_ (0.1286 g, 3.3994 mmol), and the resulting mixture was stirred for 10 min. Then, the reaction mixture was quenched with water (60 mL) and extracted with CH_2_Cl_2_ (3 × 50 mL). The organic layer was separated and washed with brine (3 × 50 mL). The organic layer was dried over anhydrous sodium sulphate, filtered, and the solvent was removed under reduced pressure, yielding a white solid as a pure product (**4**) (0.1584 g, 30% yield). ^1^H-NMR (500 MHz, CDCl_3_): δ 0.23 (s, 6H, -C*H*_3_), 0.97 (s, 9H, -C*H*_3_), 4.86 (s, 2H, -C*H*_2_), 6.46 (s, 1H, phenyl-C*H*), 6.76 (d, 1H, phenyl-C*H*, *J*_H-H_ = 8.6 Hz), 6.79 (d, 1H, phenyl-C*H*, *J*_H-H_ = 2.0 Hz), 7.36 (d, 1H, phenyl-C*H*, *J*_H-H_ = 8.6 Hz). ^13^C-NMR (125 MHz, CDCl_3_): δ −4.16, 18.24, 25.53, 60.86, 108.01, 109.14, 111.54, 117.29, 124.25, 154.04, 155.10, 159.22, 161.52. HRMS *m*/*z* calculated for C_16_H_22_NaO_4_Si ([M + Na]^+^): 329.1180, found: 329.1188.

### 2.5. Synthesis of 7-((Tert-butyldiphenylsilyl)oxy)-4-(hydroxymethyl)-2H-chromene-2-one *(**5**)*

To a solution of **3** (0.3613 g, 0.8431 mmol) in MeOH:THF (1:1) 30 mL at 0 °C was added NaBH_4_ (0.0640 g, 1.690 mmol) and the resulting mixture was stirred for 10 min. Then, the reaction mixture was quenched with water (60 mL) and extracted with dichloromethane (3 × 50 mL). The organic layer was separated and washed with brine (3 × 50 mL). The organic layer was then dried over anhydrous sodium sulphate, filtered, and the solvent was removed under reduced pressure, yielding a white solid (0.2859 g, 79% yield) as a pure product (**5**). ^1^H-NMR (500 MHz, CDCl_3_) δ 1.09 (s, 9H, -C*H*_3_), 4.78 (s, 2H, -C*H*_2_-), 6.40 (s, 1H, phenyl-C*H*), 6.69 (d, 1H, phenyl-C*H*, *J*_H-H_ = 7.6 Hz), 6.70 (s, 1H, phenyl-C*H*), 7.22 (d, 1H, phenyl-C*H*, *J*_H-H_ = 9.0 Hz), 7.37 (dd, 4H, phenyl-C*H*, *J*_H-H_ = 7.3 Hz), 7.43 (t, 2H, phenyl-C*H*, *J*_H-H_ = 7.3 Hz), 7.68 (d, 4H, phenyl-C*H*, *J*_H-H_ = 7.0 Hz). ^13^C-NMR (125 MHz, CDCl_3_) δ 19.42, 26.37, 60.76, 107.96, 109.02, 111.41, 116.98, 124.01, 128.00, 130.30, 131.71, 135.36, 154.10, 154.84, 158.97, 161.59. HRMS *m*/*z* calculated for C_26_H_26_NaO_4_Si ([M + Na]^+^): 453.1493, found: 453.1498.

### 2.6. HPLC Analysis

Qualitative HPLC analysis was performed on ZORBAX Eclipse XDB-C18 column (4.6 × 250 mm, 5 μm) with a flow rate of 1 mL/min. The mobile phase was programed to stay at 25% solvent A and 75% solvent B for 2 min, change from 25% solvent A and 75% solvent B to 5% solvent A and 95% solvent B for 10 min and stay at 5% solvent A and 95% solvent B for another 10 min, in which solvent A is 0.1% trifluoroacetic acid (TFA) in water and solvent B is 0.1% TFA in acetonitrile.

### 2.7. General Details for UV-Vis and Fluorescence Measurement

*Preparation of the stock solutions*: The stock solution of **5** was prepared by dissolving 2.7 mg of compound **5** with HPLC grade acetronitrile in a 25 mL standard volumetric flask (2.5 × 10^−4^ M). The stock solution of NaF was prepared by dissolving 10.5 mg with 1 mL deionized water (0.25 M). UV-Vis and fluorescence measurements were performed by taking appropriate amount of these stock solutions.

*UV-Vis absorption measurement*: The stock solution of **5** (80 μL) was added to the HEPES buffer solution pH = 7.4 (2.7 mL) in a 3.5 mL quartz cuvette. The UV-Vis absorption spectra were recorded before and after incubation with NaF.

*Fluorescence measurement*: The stock solution of **5** (20 μL) was added to the HEPES buffer solution pH = 7.4 (2.5 mL) in a 3.5 mL quartz cuvette. The fluorescence spectra were recorded before and after incubation with appropriate amount of NaF, using the following parameters: excitation wavelength = 375 nm, excitation slit = 15 nm, and emission slit = 5 nm. Time-dependent fluorescence changes of **5** were acquired by measuring the emission intensity at 473 nm using time-drive mode in FL WINLAB software.

These methods were also employed for compound **2**, **3**, and **4** at the same concentration.

### 2.8. Hydrolytic Stability Study

The 20 μL of the MeCN solution of compound **2**, **3**, **4**, and **5** was added to the HEPES buffer solution pH 7.4 (2.5 mL) in a 3.5 mL quartz cuvette. The fluorescence emission signals were recorded periodically using time-drive mode in FL WINLAB software. The obtained fluorescence data were used to calculate the ratios of silyl-capped coumarins in aqueous media at different time points (See the supplementary materials (SM) for the detailed kinetic data).

### 2.9. Cell Imaging Experiments

*Cell culture*: The HepG2 cell lines were grown in DMEM (Dulbecco’s Modified Eagle’s Medium) supplemented with 10% FBS (Fetal Bovine Serum) and 1% penicillin-streptomycin at 37 °C and 5% CO_2_. The cells were seeded to the 8-well Lab-tekTM II chambered coverglass (NuncTM) with the density 5 × 104 cells/well and the cells were maintained at 37 °C in a humidified 5% CO_2_ atmosphere in DMEM supplemented with 10% FBS and 1% penicillin-streptomycin for 24 h. Before the fluorescence cell imaging was performed, the cells were incubated with **5** (10 μM) for 30 min. Then, the cells were washed with phosphate-buffered saline (PBS) 3 times, and 50 µM (0.95 ppm) or 100 µM (1.90 ppm) NaF was added; the cells were then incubated for another 1 h. Finally, the cells were washed again with PBS 3 times before imaging.

*Fluorescence imaging*: Live cell imaging was performed with a Nikon A1 confocal microscope equipped with a 40× objective lens. Compound **5** was excited using a laser at 405 nm, and the emission was collected at 450 ± 25 nm.

*Cell viability assay*: In vitro cytotoxicity was measured by performing methyl thiazolyl tetrazolium (MTT) assays on the HepG2 cells. First, cells were seeded into a 96-well cell culture plate at 7 × 10^4^/well, and were cultured at 37 °C and 5% CO_2_ for 24 h; different concentrations of **5** (0, 1, 5, 10, and 20 μmol/L, diluted in DMEM) were then added to the wells. The cells were subsequently incubated for 3 h and 24 h at 37 °C under 5% CO_2_. Thereafter, MTT (5 mg/mL) was added to each well and the plate was incubated for an additional 3 h at 37 °C under 5% CO_2_. Then, the media was replaced with DMSO and the plates were shaken for 10 min before measuring the absorbance. The optical density OD570 value (Abs.) of each well, with background subtraction at 690 nm, was measured by a MG Labtech microplate reader. The following formula was used to calculate the inhibition of cell growth: Cell viability (%) = (mean of Abs. value of treatment group/mean Abs. value of control) × 100%.

## 3. Results and Discussion

### 3.1. The Synthesis of Silyl-Capped Coumarins

**Scheme 1:**
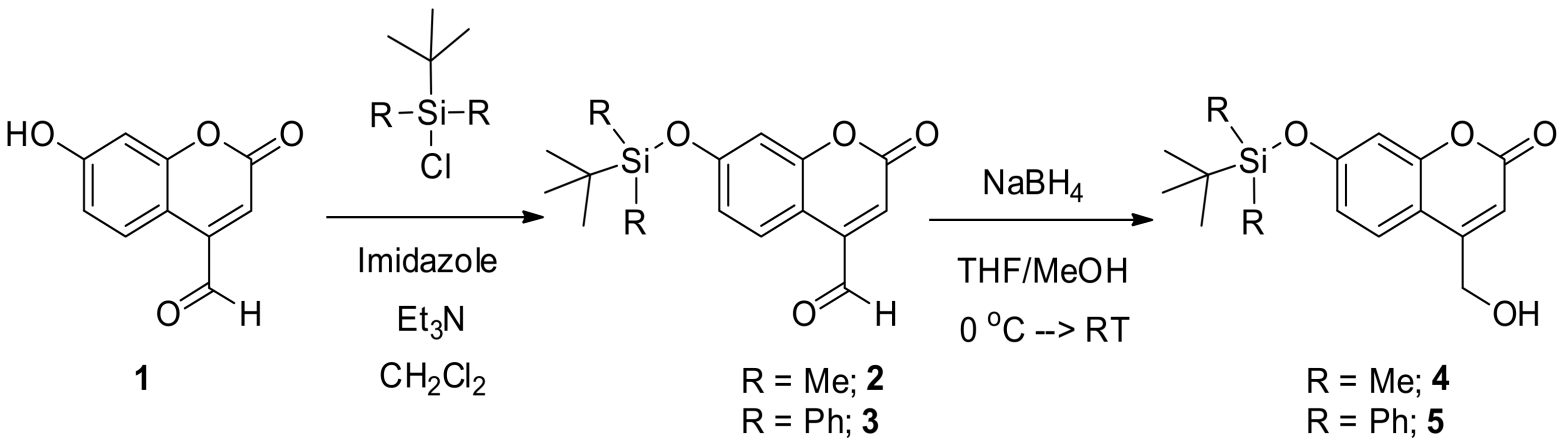
Synthesis of hydroxyl-decorated coumarins (**4** and **5**) through aldehyde-decorated coumarins (**2** and **3**).

Hydroxyl-decorated coumarins containing oxysilyl moiety (**4** and **5**) were synthesized through aldehyde-functionalized coumarin intermediates (**2** and **3**) (Scheme 1). These intermediates were prepared by silyl protection of the known 7-hydroxycoumarin-4-carbaldehyde [32] (**1**) with either *tert*-butyldimethylsilyl chloride or *tert*-butyldiphenylsilyl chloride in the presence of imidazole, and triethylamine. Afterward, the reduction of **2** and **3** with NaBH_4_ at 0 °C in THF/MeOH mixture afforded **4** and **5** in reasonable yields (30% for **4**, and 79% for **5**). All compounds were fully characterized by NMR spectroscopy (^1^H, ^13^C, and DEPT-135) and mass spectrometry. The presence of aldehyde moiety (-CHO) in **2** and **3** was confirmed by the appearance of a singlet peak in ^1^H-NMR spectra at 10.07 and 10.00 ppm and a singlet peak in ^13^C-NMR spectra at 191.91 and 191.64 ppm, respectively (Figures S1–S4 in the supplementary materials (SM). Moreover, the formation of methylene alcohol group (-CH_2_OH) in **4** and **5** were proved by the detection of a singlet peak in ^1^H-NMR spectra at 4.86 and 4.78 ppm (Figures S5 and S8 in the SM). The negative peak in DEPT-135 spectra at 60.86 and 60.76 ppm indicated the existence of -CH_2_- in **4** and **5** (Figures S7 and S10 in the SM). The detection of the molecular ions by positive-mode electrospray mass spectrometry at *m*/*z* = 327.1013 for **2**, 451.1339 for **3**, 329.1188 for **4** and 453.1498 for **5**, serves as an additional confirmation for the identity of these compounds (Figures S11–S14 in the SM). Finally, the high-performance liquid chromatography (HPLC) analysis confirmed the purity of our synthesized compounds as a single peak was observed at 6.8 min for **2**, 10.3 min for **3**, 5.9 min for **4**, and 10.1 min for **5** in the UV-HPLC profiles (Figures S15–S18 in the SM).

### 3.2. UV-Visible Spectra Responses of the Synthesized Coumarins toward Fluoride

**Scheme 2:**
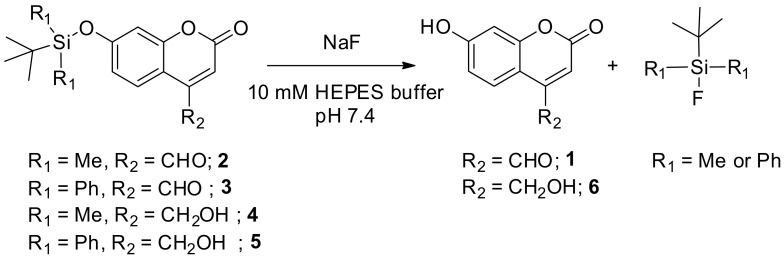
The fluoride-promoted Si-O bond cleavage reaction of silyl-capped coumarins synthesized in this work.

**Figure 2:**
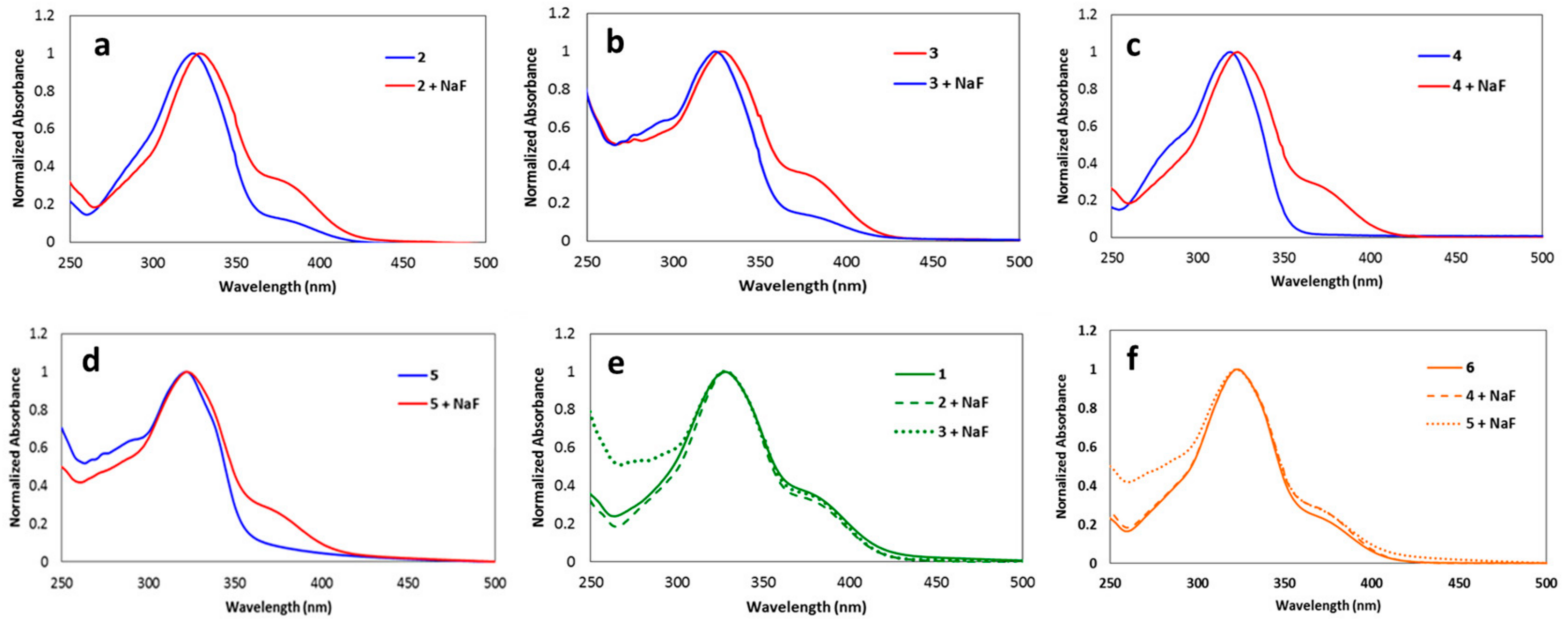
(**a**–**d**) The normalized UV-Vis absorption spectra of **2** (**a**), **3** (**b**), **4** (**c**), and **5** (**d**) (7.2 µM) in HEPES buffer pH 7.4 (contain 3% MeCN) before (blue line) and after (red line) incubation with NaF (12.5 mM) for 1 h (the absolute absorption spectra are shown in Figures S19–S22 in the SM) and (**e**,**f**) the UV-Vis absorption spectra of **2**, **3**, **4**, and **5** (7.2 µM) in HEPES buffer pH 7.4 (contain 3% MeCN) after incubation with NaF (12.5 mM) for 1 h compared with the UV-Vis absorption spectra of the corresponding non-silylated coumarins **1** (**e**) and **6** (**f**).

Next, UV-Visible spectroscopy was used to investigate the reaction of these coumarin derivatives with the fluoride ions (Scheme 2). As shown in Figure 2a–d, the new shoulder peak at 375 nm appeared in UV-Vis absorption spectrum of compound **2**, **3**, **4**, and **5** (7.2 µM) after incubation with sodium fluoride (12.5 Mm) in HEPES buffer pH 7.4 (contain 3% MeCN) for 1 h. We also noticed that the absorption spectra of compound **2**, **3**, **4**, and **5** after the addition of fluoride are similar to those of corresponding non-silylated coumarin derivatives (compound **1** and **6**) in the same solvent system (Figure 2e,f). These results suggested that all derivatives underwent Si-O bond cleavage upon addition of fluoride like other reported silyl-capped coumarin derivatives [27,28,29,30,31].

### 3.3. Fluorescence Response of the Synthesized Coumarins toward Fluoride

**Figure 3:**
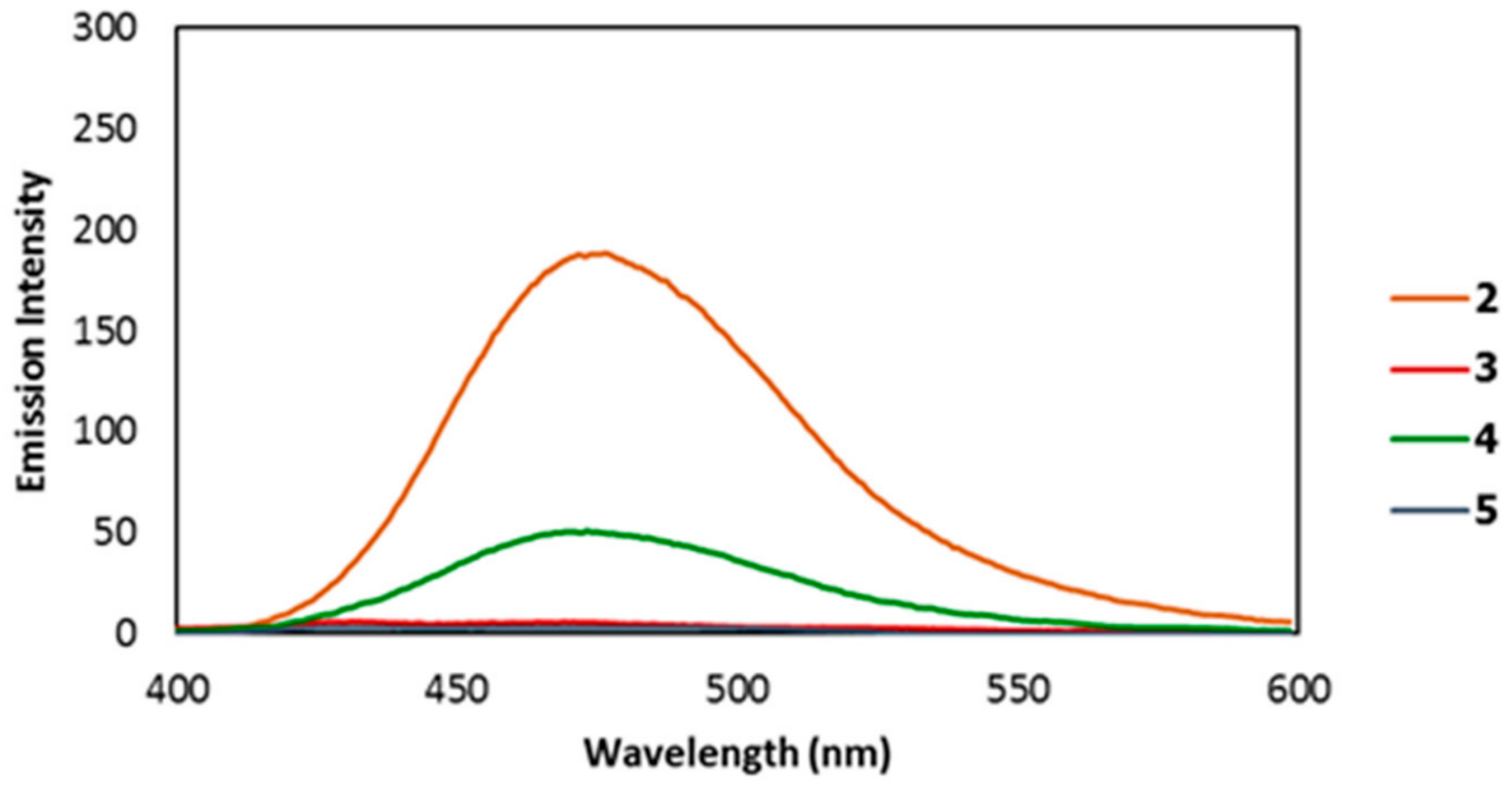
The fluorescence emission spectra of compound **2**, **3**, **4**, and **5** (2 μM) obtained immediately after dissolving in HEPES buffer solution pH 7.4 (contain 0.8% MeCN).

Then, the emission properties of the synthesized coumarins were examined by fluorescence spectroscopy. As **2**, **3**, **4**, and **5** do not fully dissolve in 100% HEPES buffer solution, a trace amount of MeCN (20 μL) was included in the aqueous media (2.5 mL) for the fluorescence measurements. However, it is important to point out that the percentage of MeCN in the resulting aqueous media was only 0.8% (See materials and methods section). Upon excitation at 375 nm, compound **2** and **4** immediately demonstrated a strong fluorescence enhancement maximum at 475 nm and 473 nm, respectively, in HEPES buffer solution pH 7.4 (contain 0.8% MeCN) without fluoride (Figure 3). On the other hand, compound **3** and **5** showed a weak fluorescence intensity in the same solvent system. These findings were previously observed for compound **V** and **VI** (Figure 1) in the previous publication [27], and were ascribed to stronger Si-O bond polarizations in the coumarin derivatives containing *tert*-butyldimethyloxysilyl moiety (**V**, **2**, and **4**) in aqueous solution imitating intramolecular charge transfer (ICT) phenomena without desilylation.

**Figure 4:**
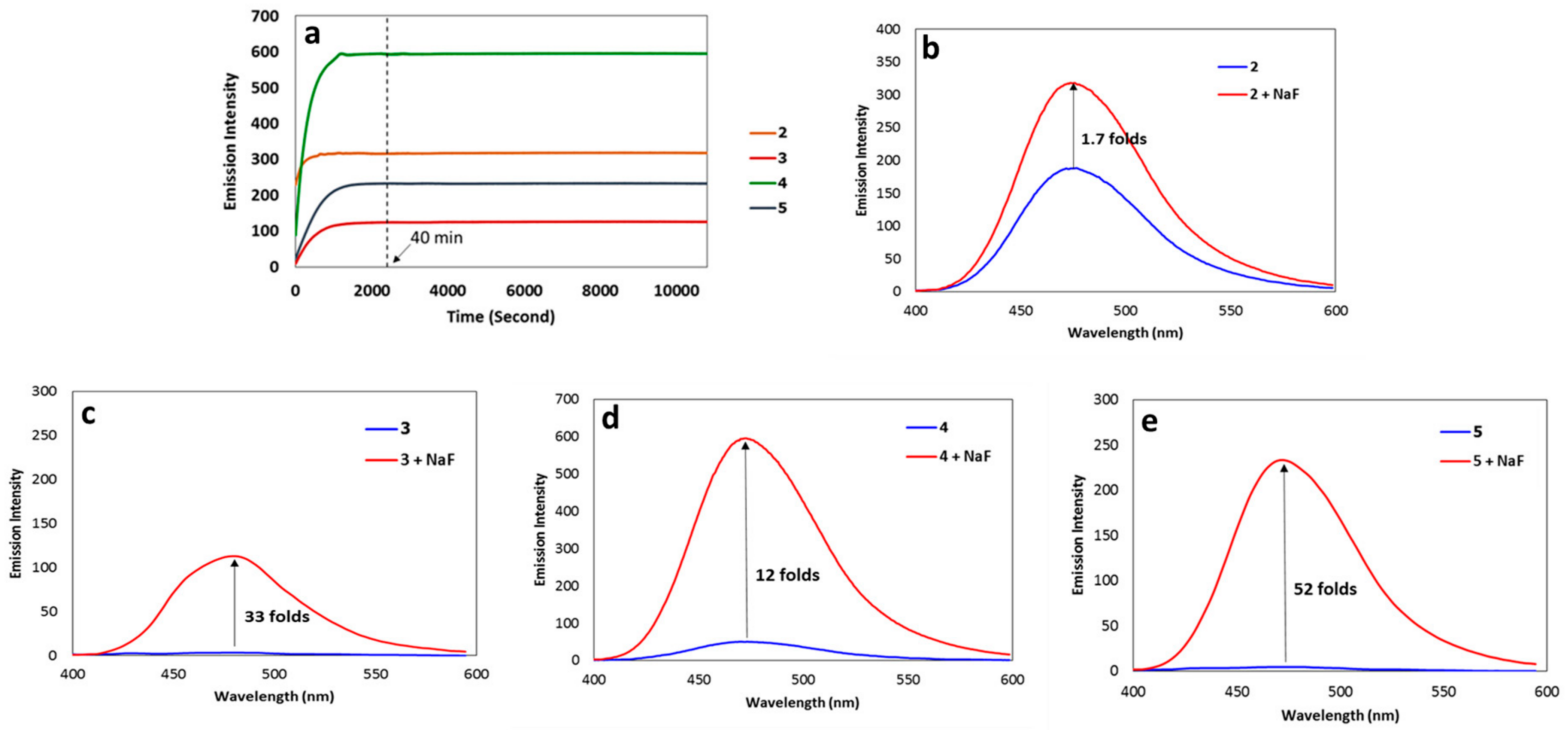
(**a**) Time courses of fluoride-promoted Si-O bond cleavage reaction of **2**, **3**, **4**, and **5** (2 μM) after addition of NaF (1 mM) in HEPES buffer solution pH 7.4 (contain 0.8% MeCN) and (**b**–**e**) Fluorescence spectral changes of **2** (**b**), **3** (**c**), **4** (**d**), and **5** (**e**) (2 μM) before (blue line) and after (red line) addition of NaF (1 mM) at 3 h incubation time in HEPES buffer solution pH 7.4 (contain 0.8% MeCN).

The fluoride-promoted Si-O bond cleavage reactions were also studied by fluorescence measurements. The changes in fluorescence emission signals of compound **2**, **3**, **4**, and **5** (2 μM) after the addition of excess amount of NaF (1 mM) in HEPES buffer solution pH 7.4 (contain 0.8% MeCN) were monitored at 475 nm in the case of **2** and **3**, and 473 nm in the case of **4** and **5**; the results are shown in Figure 4a. Based on these data, the fluorescence emission intensity of all compounds rapidly increased right after the exposure to NaF (1 mM), and remained constant at around 35–40 min incubation time. Compound **5** exhibited the highest fluorescence enhancement (52 folds), followed by compound **3** (33 folds), **4** (12 folds), and **2** (1.7 folds), respectively (Figure 4b–e).

### 3.4. Hydrolytic Stabilities of the Silyl-Capped Coumarins

**Scheme 3:**
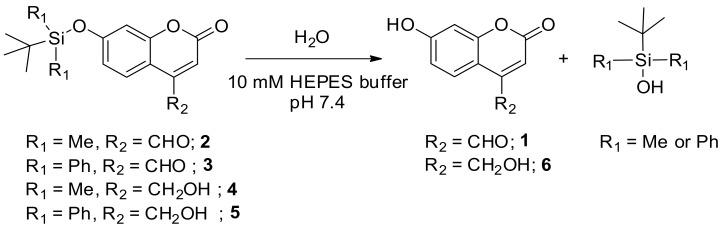
The hydrolytic reaction of silyl-capped coumarin (**2**, **3**, **4**, and **5**) in aqueous solution.

**Figure 5:**
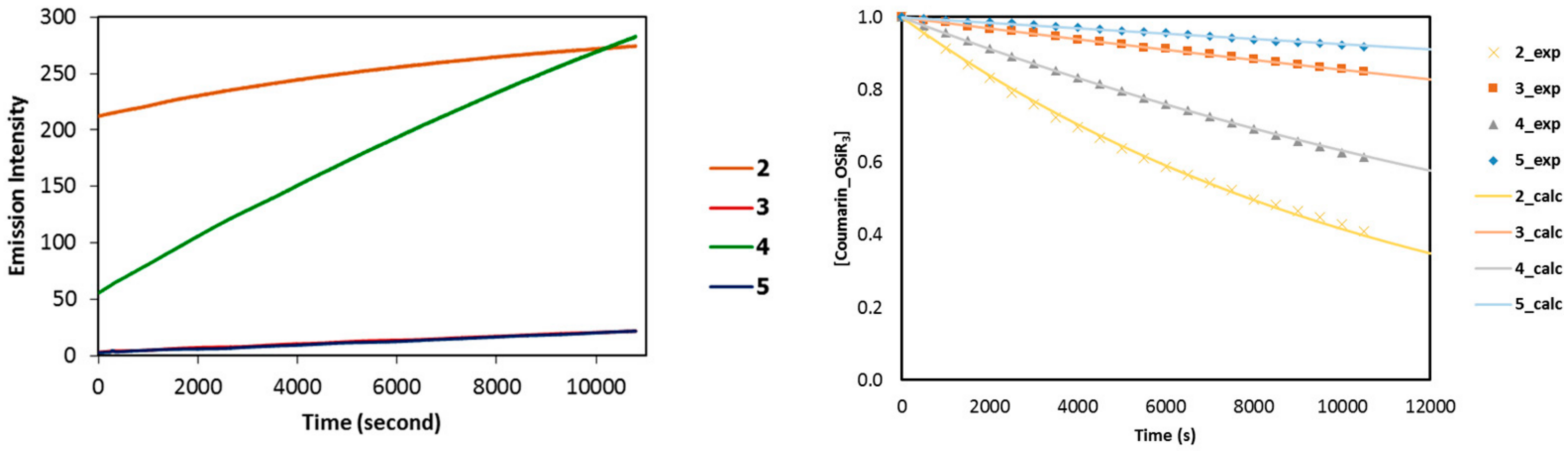
(**Left**) Fluorescence signal changes upon dissolving **2**, **3**, **4**, and **5** (2 μM) in HEPES beffer solution pH 7.4 (contain 0.8% MeCN) for 3 h. (**Right**) Kinetic plots for the hydrolysis of **2**, **3**, **4**, and **5** (2 μM) in HEPES buffer solution pH 7.4 (contain 0.8% MeCN).

As these silyl-capped coumarin derivatives are subjected to detecting fluoride ions in water samples, we decided to evaluate hydrolytic stability in aqueous systems using a method adapted from the reported hydrolytic stability study of BF_3_- and PF_5_- containing compounds [34,35]. The hydrolysis reactions, which convert the silyl-capped coumarins into the corresponding desilylated products (Scheme 3) according to a first-order rate equation (*ν* = k_obs_[Coumarin_OSiR_3_]), were monitored by fluorescence spectrometer. The fluorescence signals detected after incubation of **2**, **3**, **4**, and **5** (2 μM) in HEPES buffer pH 7.4 (contain 0.8% MeCN) at each time point (Figure 5 (Left)), comparative with the maximum fluorescence intensities (F_max_) obtained after the incubation of **2**, **3**, **4**, and **5** (2 μM) with an excess amount of NaF (1 mM) (Figure 4a) in the same solvent system were related to the ratio of silyl-capped coumarin (Coumarin_OSiR_3_) in aqueous media (See the SM for the detailed kinetic data). The kinetic plots shown in Figure 5 (Right) suggest that compound **3** (k_obs_ = 1.56 × 10^−5^ s^−1^) and **5** (k_obs_ = 7.70 × 10^−6^ s^−1^) are more hydrolytically stable in aqueous systems than compound **2** (k_obs_ = 8.76 × 10^−5^ s^−1^) and **4** (k_obs_ = 4.58 × 10^−5^ s^−1^). This phenomenon could be the results from less water accessibility of the silicon center in **3** and **5**, due to the bulkiness of the *tert*-butyldiphenyloxysilyl moiety. As compound **5** was the most stable derivative in aqueous solution, and exhibited the highest fluorescence enhancement in this series, it was selected for further studies as a fluoride sensor.

### 3.5. Solubility, Selectivity, and Interference Studies of Probe ***5***

**Figure 6:**
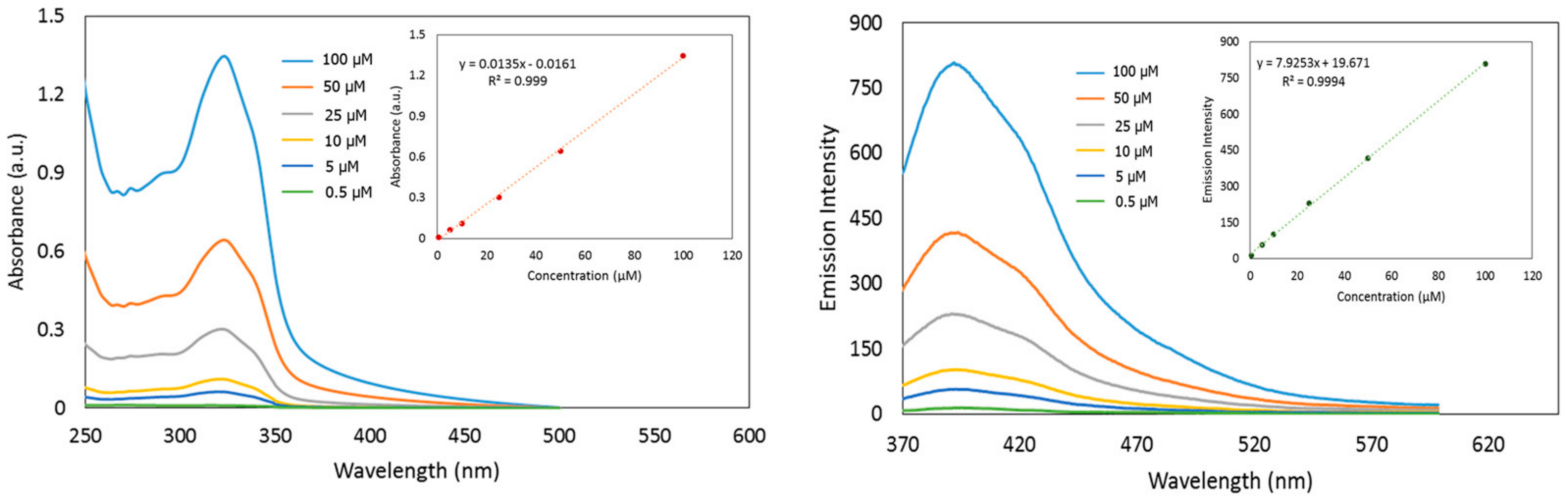
(**Left**) Absorption spectra of **5** at different concentrations (0.5, 5, 10, 25, 50, 100 μM) in HEPES buffer solution pH 7.4 (contain 0.8% MeCN). The inset displays the linear relationship between the absorbances at 323 nm and the concentrations of **5**. (**Right**) Fluorescence spectra of **5** (excited at 350 nm) at different concentrations (0.5, 5, 10, 25, 50, 100 μM) in HEPES buffer solution pH 7.4 (contain 0.8% MeCN). The inset displays the linear relationship between the emission intensities at 393 nm and the concentrations of **5**.

First, we examined the solubility of the hydroxyl-decorated coumarin (**5**) in our aqueous system (HEPES buffer solution pH 7.4 containing 0.8% MeCN) by UV-visible and fluorescence spectroscopic techniques. As shown in Figure 6, the absorbances at 323 nm (Figure 6 (Left)) and the emission intensities at 393 nm (Figure 6 (Right)) of probe **5** demonstrated the gratifying linear regressions (R^2^ = 0.999) with the concentrations of **5** ranging from 0.5 μM to 100 μM. These results clearly indicated that compound **5** is soluble in HEPES buffer solution pH 7.4 containing 0.8% MeCN at the concentrations up to 100 μM without aggregation effects.

**Figure 7:**
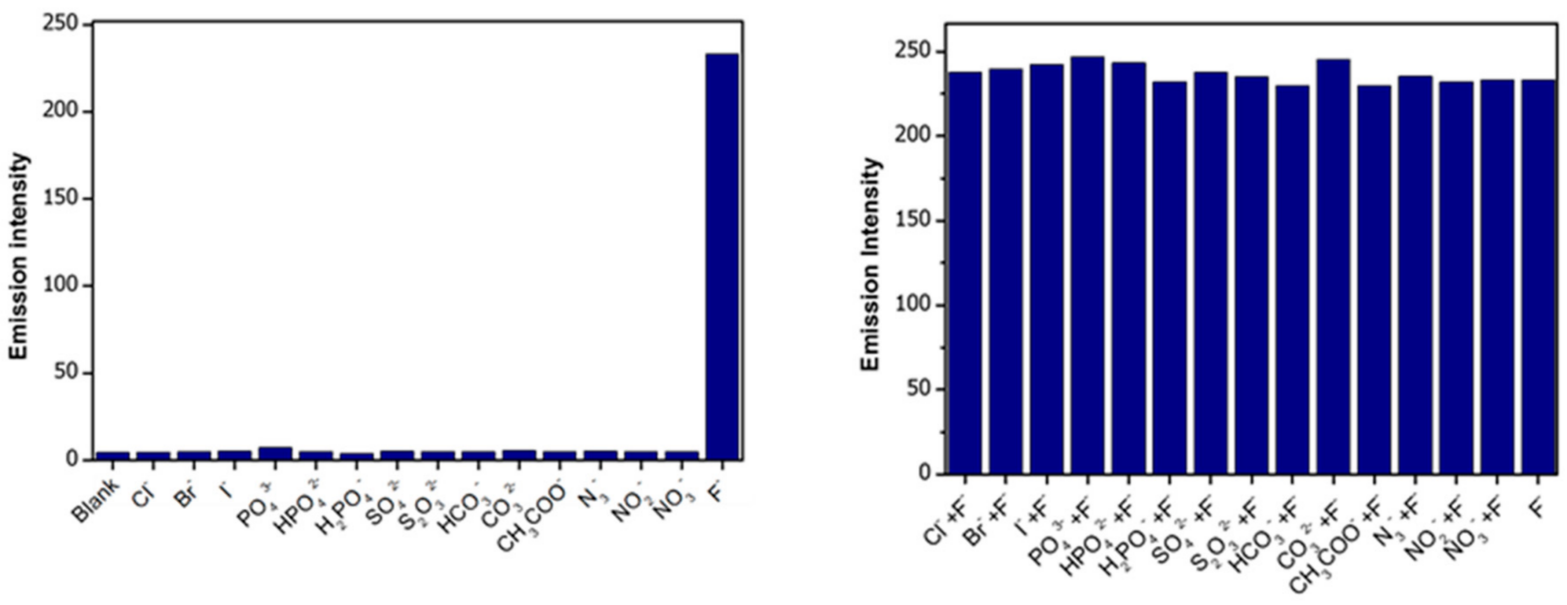
(**Left**) Bar chart demonstrating fluorescence intensities at 473 nm for **5** (2 μM) in the presence of different anions (1 mM). The fluorescence spectra corresponding to this chart are displayed in Figure S27. (**Right**) Bar chart demonstrating fluorescence intensities at 473 nm for **5** (2 μM) in the presence of fluoride (1 mM) co-existing with other anions (1 mM). The fluorescence spectra corresponding to this chart are displayed in Figure S28.

Subsequently, we assessed the selectivity of probe **5** toward fluoride (F^−^) in HEPES buffer solution pH 7.4 (contain 0.8% MeCN). As demonstrated in Figure 7 (Left), only F^−^ induced a noticeable fluorescence intensity enhancement at 473 nm, while the addition of other anions including, Cl^−^, Br^−^, I^−^, PO_4_^3−^, HPO_4_^2−^, H_2_PO_4_^−^, SO_4_^2−^, S_2_O_3_^2−^, HCO_3_^−^, CO_3_^2−^, CH_3_COO^−^, N_3_^−^, NO_2_^−^, and NO_3_^−^ showed almost no change in fluorescence intensity. In addition, the effects of interference of the above-mentioned analytes on F^−^ sensing were investigated (Figure 7 (Right)). The results clearly indicated that the co-existenceof these anions does not interfere with the reaction of fluoride with the chemical sensor **5**, as fluorescence signals were reasonably increased. These outcomes confirmed a high selectivity of compound **5** toward F^−^ without any interfering effects from other anions.

### 3.6. Fluorescence Response and Limit of Detection of Probe ***5*** Toward Fluoride

**Figure 8:**
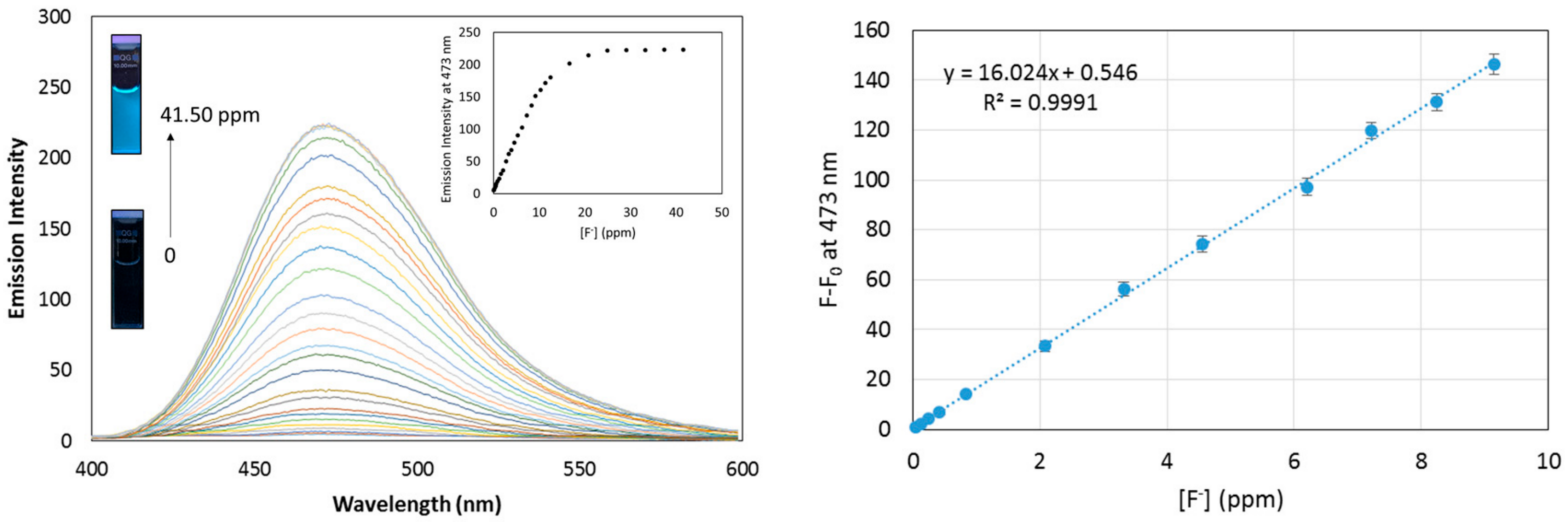
(**Left**) Fluorescence spectra of **5** (2 μM) upon incubation with various amounts of fluoride (final concentration: 0, 0.04, 0.12, 0.24, 0.42, 0.60, 0.82, 1.24, 1.60, 2.08, 2.72, 3.32, 3.86, 4.56, 5.32, 6.20, 7.22, 8.25, 9.15, 10.25, 11.32, 12.48, 16.63, 20.78, 24.90, 29.10, 33.20, 37.35, 41.50 ppm) in HEPES buffer solution pH 7.4 (contain 0.8% MeCN) at 40 min incubation time. (**Right**) Standard calibration curve demonstrating the fluorescence intensity increment at 473 nm (*n* = 3) as a function of fluoride concentrations (0.04, 0.12, 0.24, 0.42, 0.83, 2.08, 3.32, 4.56, 6.21, 7.22, 8.25, 9.15 ppm) incubated with **5** (2 μM) in HEPES buffer solution pH 7.4 (contain 0.8% MeCN) for 40 min.

The fluorescence spectra of **5** upon addition of varied amounts of fluoride (0.04–41.50 ppm) in HEPES buffer solution pH 7.4 (contain 0.8% MeCN) acquired at 40 min incubation time are displayed in Figure 8 (Left). These spectra clearly demonstrate that the fluorescence emission of probe **5** gradually increases with the addition of fluoride. However, a satisfactory linear relationship between emission intensity increment and fluoride concentrations was only observed in the range from 0 to 9.15 ppm of fluoride concentrations with the regression equation y = 16.024x + 0.546, as shown in Figure 8 (Right). Based on this standard calibration curve, the limit of detection (LOD) of probe **5** was determined as 43 ppb by 3σ/*m* formula, where σ is the standard deviation of blank samples and *m* is the slope of the calibration curve (See the SM). This working concentration range and the LOD are suitable for fluoride detection at the standard value of fluoride concentration (0.7 ppm) in drinking water recommended by U.S. Department of Health and Human Services [36].

As shown in the comparison table with other reported coumarin-based probes (Table 1), probe **5** can detect fluoride in the solvent system with the lowest portion of organic co-solvent (0.8% MeCN) compared to probe **I**, **II**, **III**, and **IV** and with low limit of detection compared to probe **II**. Although probe **I** and **III** exhibited extremely low limit of detection (0.8 and 2.1 ppb), they were tested in the system containing high portion of organic solvent (70% Acetone for **I** and 100% MeCN for **III**) with less hydration and fewer solubility effects.

### 3.7. Fluoride Sensing in Collected Water Samples

Compound **5** was then employed to determine fluoride (F^−^) content in tap water and river water samples. As displayed in Table 2, this molecular sensor accurately detected fluoride ions in the collected samples spiked with 0.5 and 5.0 ppm fluoride concentrations with the recovery close to 100%. These results confirmed the satisfactory analytical accuracy (<5%) error of this method. Additionally, the fact that the compositions of tap water and river water do not significantly interfere with fluoride detection suggested the potential of utilizing this compound in water quality monitoring applications.

### 3.8. Imaging of Fluoride in Live Cells

**Figure 9:**
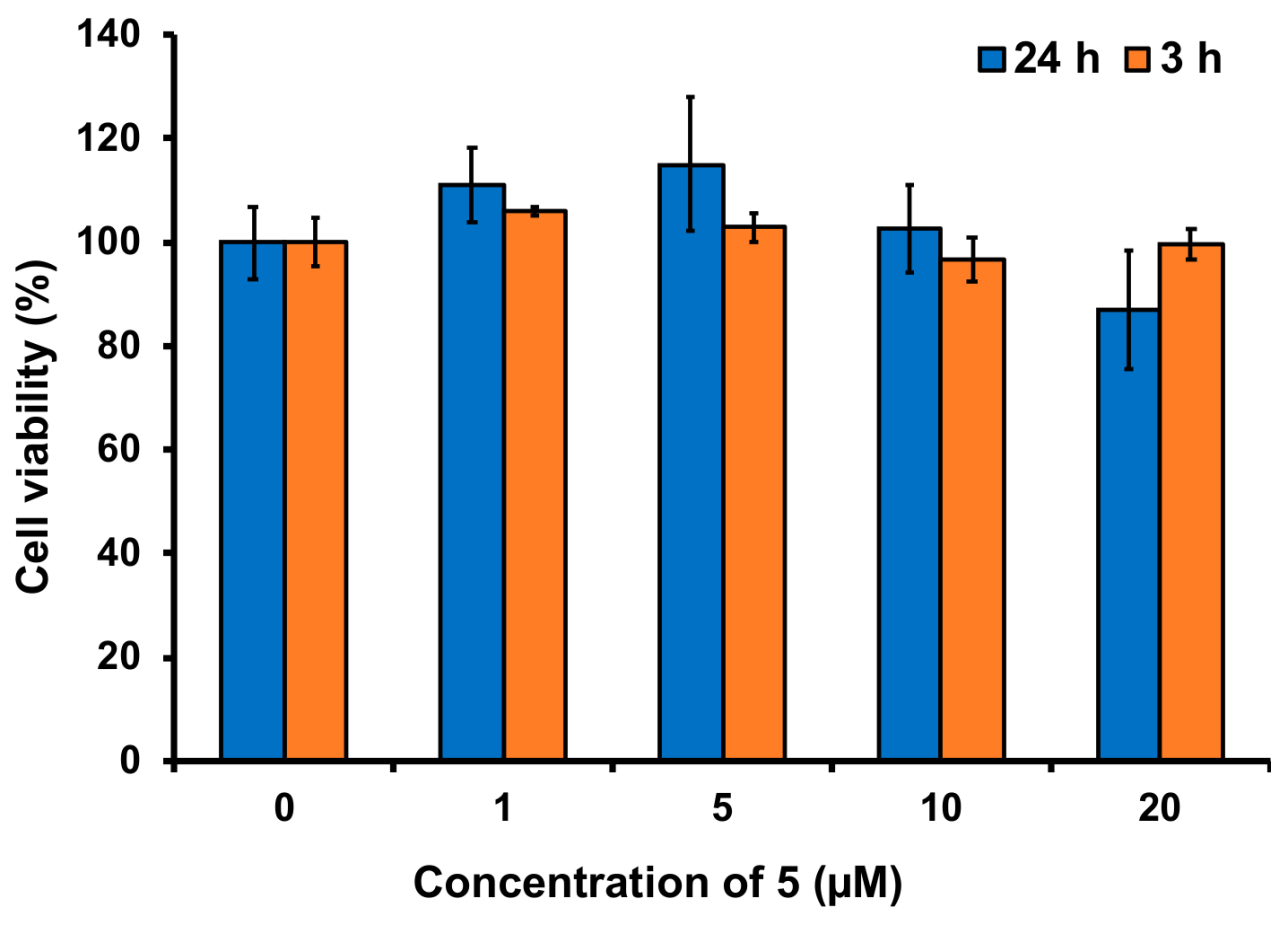
Cell viability values (%) estimated by MTT proliferation test versus incubation concentrations of **5**. HepG2 cells were cultured in the presence of **5** (1–20 μM) at 37 °C for 3 h (orange bars) and 24 h (blue bars).

In the last section of this work, we investigated the ability of compound **5** to image fluoride ions in living matrices. As fluoride was linked to the appearance of a rare form of liver cancer in mice [37], the liver cancer cell line (HepG2 cells) was selected as a model for fluoride imaging experiment. Compound **5** was confirmed to be non-toxic to HepG2 cells at levels up to 20 μM, as shown in Figure 9. These results imply that **5** is suitable for live cell imaging within 20 μM. Therefore, we chose concentration at 10 μM for future experiments.

**Figure 10:**
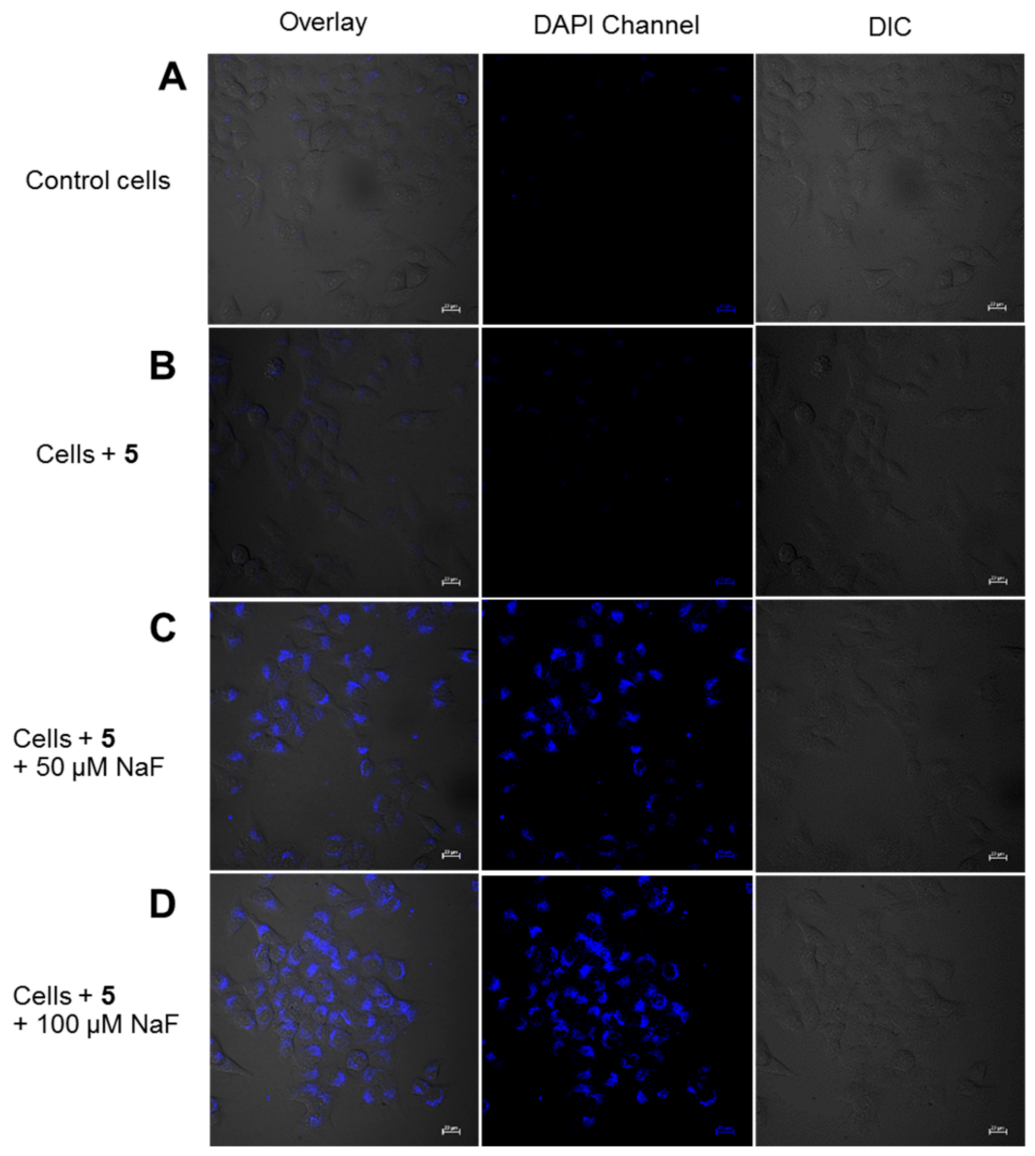
(**A**–**D**) Confocal laser scanning microscopy (CLSM) images of living HepG2 cells. Panel A, control cells. Panel B, cells incubated with **5** (10 μM) for 30 min and subsequently 1 h without F^−^ ions. Panel C, cells incubated with **5** (10 μM) for 30 min and subsequently incubated with NaF (50 µM) for 1 h. Panel D, cells incubated with **5** (10 μM) for 30 min and subsequently incubated with NaF (100 µM) for 1 h (for compound **5**: λ_ex_= 405 nm, λ_em_ = 450 ± 25 nm, scale bars = 20 µm). All images were collected at the same microscopy settings.

**Figure 11:**
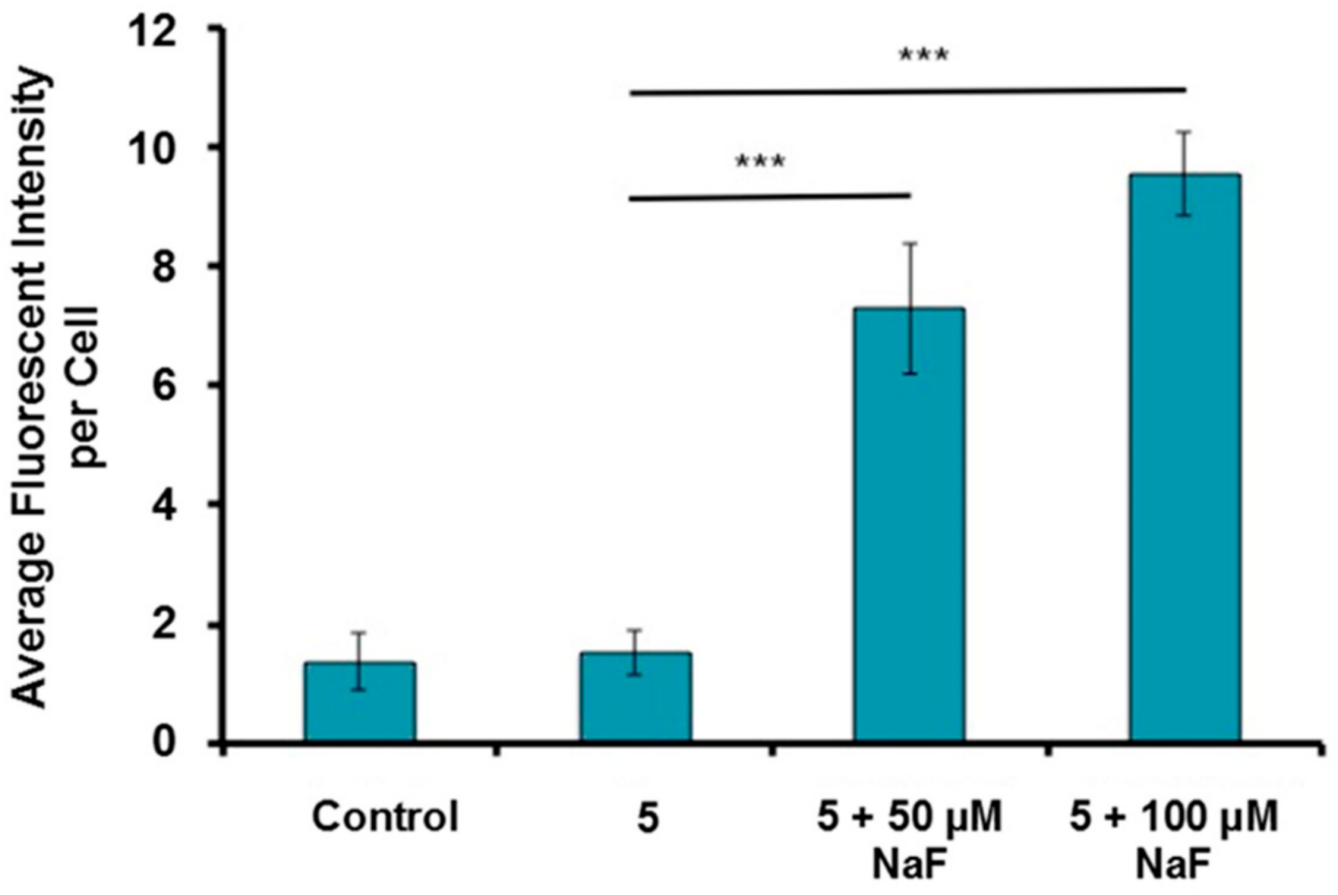
Average fluorescent intensity per cell from each group of the cell imaging study (average values and error bars are acquired from 3 images of each imaging condition). Statistical analysis was calculated by student’s *t*-test (*** *p* < 0.001).

Next, to ensure the application in cancer cells imaging, HepG2 cells were incubated with compound **5** (10 μM) for 30 min under the physiological conditions, followed by washing with phosphate buffer solution (PBS), and incubating with different concentrations of fluoride for 1 h, consecutively. The cells incubated with 50 μM and 100 μM of NaF demonstrated a distinct fluorescence signal (Figure 10C,D) compared to the control experiment (Figure 10A,B). Moreover, average fluorescent intensity per cell for representative cells from each group (20 cells) clearly demonstrated the signal differences between the treated groups and the control groups (Figure 11). These results indicated that compound **5** can penetrate the cell membrane, retain in the cells, and efficiently image fluoride in cellular system pointing its capability for investigating fluoride toxicity and bioactivity in biological systems.

## 4. Conclusions

In summary, we have synthesized new coumarin-based fluorescent probes for fluoride detection relying on a fluoride-promoted desilylation triggering fluorogenic reaction in aqueous media. These derivatives exhibited different fluorescent responses toward fluoride, and different hydrolytic stabilities depending on the nature of the substituents on the silicon atom and coumarin backbone. The hydroxyl-decorated coumarin linked with *tert*-butyldiphenyloxysilyl group (**5**) possessed the highest fluorescence enhancement toward fluoride, and was the most hydrolytically stable in this series. This compound also exhibited high fluoride-selectivity over several anions, and a satisfactory limit of detection at 0.043 ppm (43 ppb), which corresponds to the standard value of fluoride concentration (0.7 ppm) in drinking water [36]. Importantly, this compound was successfully employed in the detection fluoride levels in the fluoride-spiked water samples, such as tap water and river water, with significant analytical accuracy (less than 5% error). Finally, probe **5** was efficiently used in fluorescence bioimaging of fluoride in HepG2 cells, suggesting its excellent cell permeability, ability to retain inside the living cells, and good stability under physiological conditions.

## Figures and Tables

**Table 1:** Comparison of fluorescent probe **5** with other reported coumarin-based probes used for the detection of fluoride.

Probe	Solvent System	Limit of Detection	Reference
**I**	Acetone-water (70% Acetone *v*/*v*)	0.8 ppb	[28]
**II**	THF-water (4% THF *v*/*v*)	200 ppb	[29]
**III**	MeCN	2.1 ppb	[30]
**IV**	MeOH-HEPES buffer solution (50% MeOH *v*/*v*)	N/D	[31]
**VI**	HEPES buffer solution	N/D	[27]
**5**	MeCN-HEPES buffer solution (0.8% MeCN *v*/*v*)	43 ppb	This work

N/D = Not Determined.

**Table 2:** Determination of the fluoride anion in tap water and river water samples (*n* = 3) ^[a]^.

Samples ^[b]^	Amount of Fluoride (ppm)		Recovery (%)
	**Added**	**Found**	
Tap water	0.50	0.51 ± 0.02	102.7 ± 4.2
	5.0	4.82 ± 0.08	96.4 ± 1.5
River water ^[c]^	0.50	0.52 ± 0.03	104.0 ± 5.3
	5.0	4.87 ± 0.07	97.3 ± 1.3

^[a]^ The measurements were recorded 40 min after the addition of fluoride. ^[b]^ The tap water and river water samples were diluted 2-fold and 10-fold, respectively, by the addition of 10 mM HEPES buffer solution (pH 7.4). ^[c]^ The river water samples were filtered through celite before measurements.

## Supplementary Materials

The following are available online at http://www.mdpi.com/1424-8220/18/7/2042/s1, Figure S1: ^1^H-NMR spectra of compound **2**, Figure S2: ^13^C-NMR spectra of compound **2**, Figure S3: ^1^H-NMR spectra of compound **3**, Figure S4: ^13^C-NMR spectra of compound **3**, Figure S5: ^1^H-NMR spectra of compound **4**, Figure S6: ^13^C-NMR spectra of compound **4**, Figure S7: DEPT-135 NMR spectra of compound **4**, Figure S8: ^1^H-NMR spectra of **5**, Figure S9: ^13^C-NMR spectra of **5**, Figure S10: DEPT-135 NMR spectra of **5**, Figure S11: High resolution mass spectra of compound **2**, Figure S12: High resolution mass spectra of compound **3**, Figure S13: High resolution mass spectra of compound **4**, Figure S14: High resolution mass spectra of compound **5**, Figure S15: UV-HPLC profiles of compound **2**, Figure S16: UV-HPLC profiles of **3**, Figure S17: UV-HPLC profiles of **4**, Figure S18: UV-HPLC profiles of **5**, Figure S19: Absolute absorption spectra of **2** (7.2 μM) n HEPES buffer pH 7.4 (contain 3% MeCN) before (blue line) and after (red line) incubation with NaF (12.5 mM) for 1 h, Figure S20: Absolute absorption spectra of **3** (7.2 μM) in HEPES buffer pH 7.4 (contain 3% MeCN) before (blue line) and after (red line) incubation with NaF (12.5 mM) for 1 h, Figure S21: Absolute absorption spectra of **4** (7.2 μM) in HEPES buffer pH 7.4 (contain 3% MeCN) before (blue line) and after (red line) incubation with NaF (12.5 mM) for 1 h, Figure S22: Absolute absorption spectra of **5** (7.2 μM) in HEPES buffer pH 7.4 (contain 3% MeCN) before (blue line) and after (red line) incubation with NaF (12.5 mM) for 1 h, Figure S23: Fluorescence signal changes upon dissolving **2** (2 μM) in HEPES buffer solution pH 7.4 (contain 0.8% MeCN) monitored for 3 h (Solid line) and the fluorescence signal changes upon addition of excess amount of NaF (1 mM) into the solution of **2** (2 μM) in HEPES buffer solution pH 7.4 (contain 0.8% MeCN) monitored for 3 h (dashed line), Figure S24: Fluorescence signal changes upon dissolving **3** (2 μM) in HEPES buffer solution pH 7.4 (contain 0.8% MeCN) monitored for 3 h (Solid line) and the fluorescence signal changes upon addition of excess amount of NaF (1 mM) into the solution of **3** (2 μM) in HEPES buffer solution pH 7.4 (contain 0.8% MeCN) monitored for 3 h (dashed line), Figure S25: Fluorescence signal changes upon dissolving **4** (2 μM) in HEPES buffer solution pH 7.4 (contain 0.8% MeCN) monitored for 3 h (Solid line) and the fluorescence signal changes upon addition of excess amount of NaF (1 mM) into the solution of **4** (2 μM) in HEPES buffer solution pH 7.4 (contain 0.8% MeCN) monitored for 3 h (dashed line), Figure S26: Fluorescence signal changes upon dissolving **5** (2 μM) in HEPES buffer solution pH 7.4 (contain 0.8% MeCN) monitored for 3 h (Solid line) and the fluorescence signal changes upon addition of excess amount of NaF (1 mM) into the solution of **5** (2 μM) in HEPES buffer solution pH 7.4 (contain 0.8% MeCN) monitored for 3 h (dashed line), Figure S27: The fluorescence spectra of **5** (2 μM) in the presence of different anions (1 mM) in HEPES buffer solution pH 7.4 (contain 0.8% MeCN), Figure S28: The fluorescence spectra of **5** (2 μM) in the presence of fluoride (1 mM) co-existing with other anions (1 mM) in HEPES buffer solution pH 7.4 (contain 0.8% MeCN), Table S1. Kinetic data for the hydrolysis of **2**, Table S2. Kinetic data for the hydrolysis of **3**, Table S3. Kinetic data for the hydrolysis of **4**, Table S4. Kinetic data for the hydrolysis of **5**.
