# Supplementary material for: Coumarin Probe for Selective Detection of Fluoride Ions in Aqueous Solution and Its Bioimaging in Live Cells
Source: Sensors (Basel). 2018 Jun 26;18(7):2042. doi: 10.3390/s18072042 (PMC6069086; doi:10.3390/s18072042)
Supplement: Supplementary file 1 [file sensors-18-02042-s001.pdf]

Supporting Information

Coumarin probe for selective detection of fluoride ion in aqueous solution and its bioimaging in live cells

Kantapat Chansaenpak,*[a] Anyanee Kamkaew,[b] Oratai Weeranantanapan,[c] Khomson Suttisintong,[a] Gamolwan Tumcharern[a]

[a]National Nanotechnology Center, National Science and Technology Development Agency, Thailand Science Park, Pathum Thani, Thailand 12120.

[b]School of Chemistry, Institute of Science, Suranaree University of Technology, Nakhon Ratchasima, Thailand 30000

[c]School of Preclinical Science, Institute of Science, Suranaree University of Technology, Nakhon Ratchasima, Thailand 30000

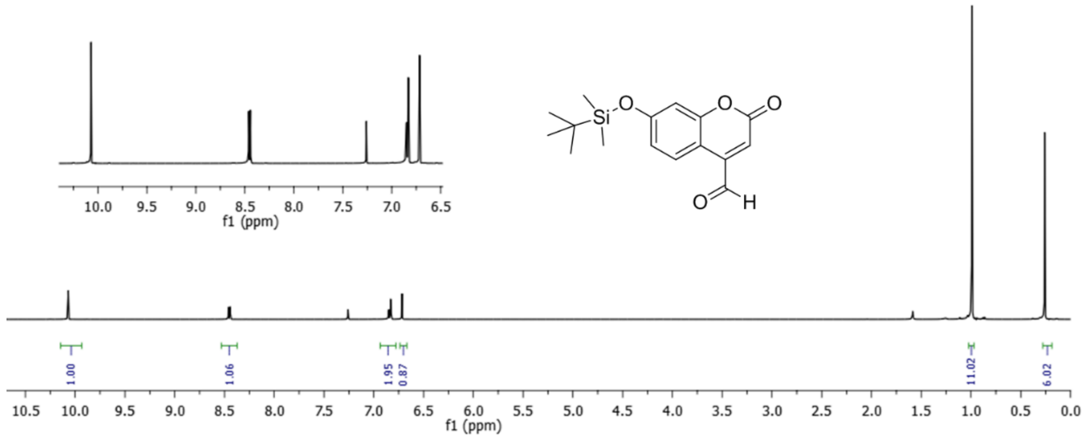

Figure S1. ^{1}H NMR spectrum of **2**

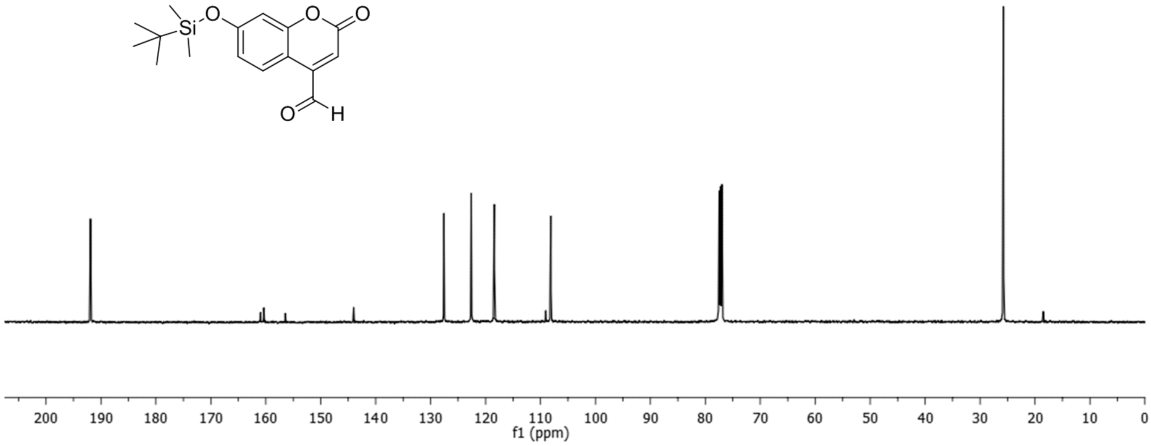

Figure S2. ^{13}C NMR spectrum of **2**

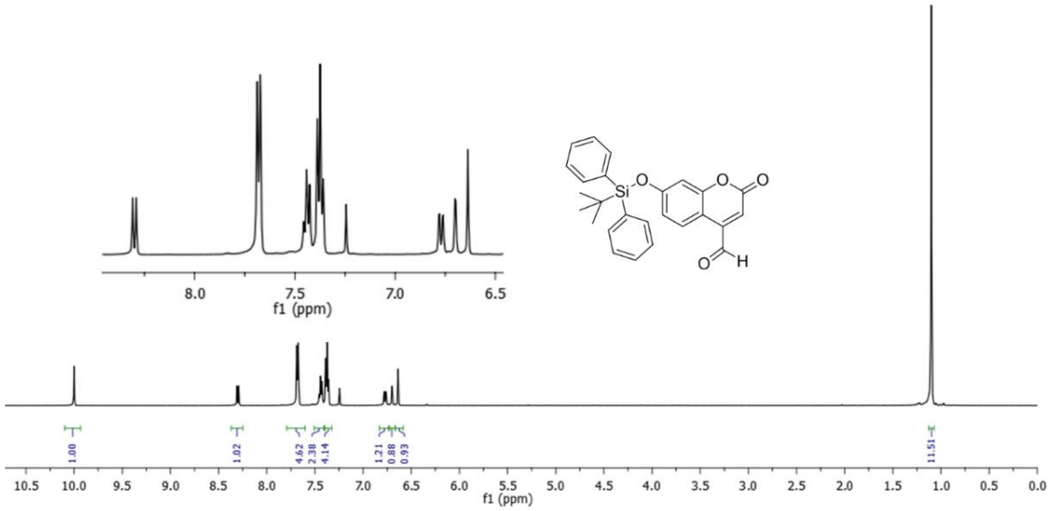

Figure S3. ^{1}H NMR spectrum of **3**

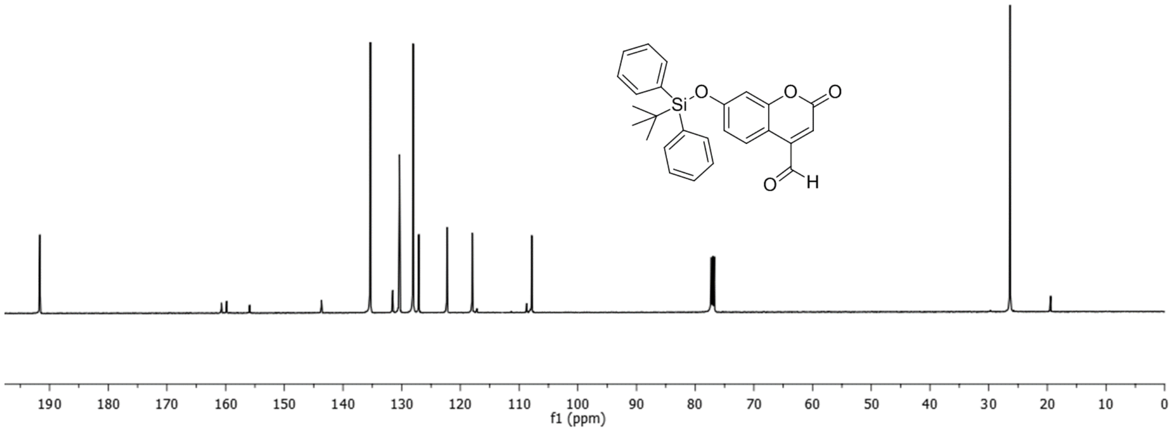

Figure S4. ^{13}C NMR spectrum of **3**

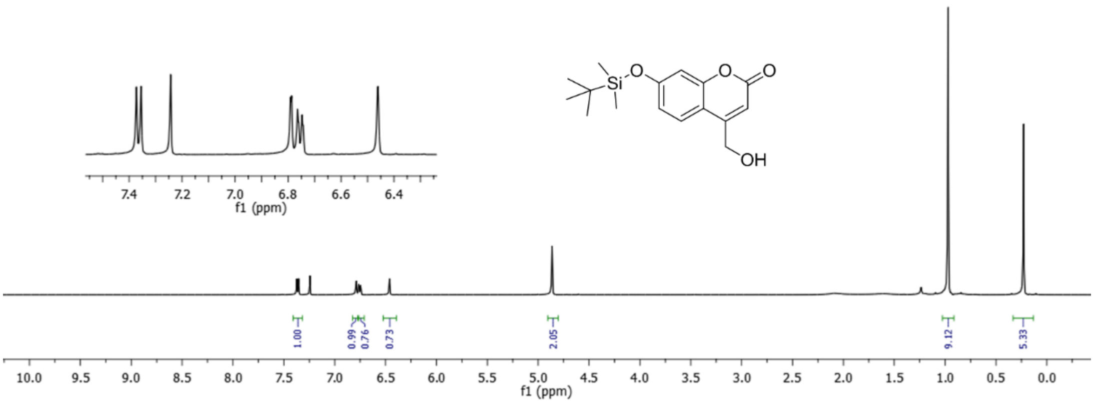

Figure S5. ^{1}H NMR spectrum of **4**

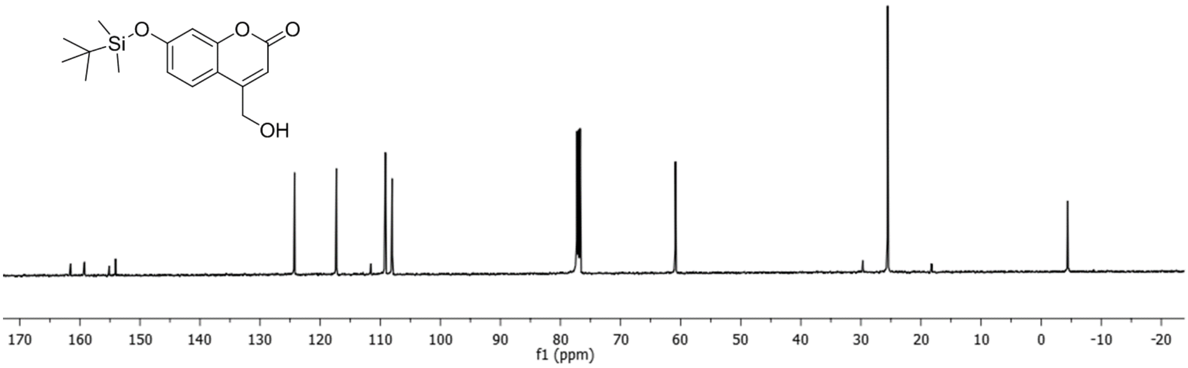

Figure S6. ^{13}C NMR spectrum of **4**

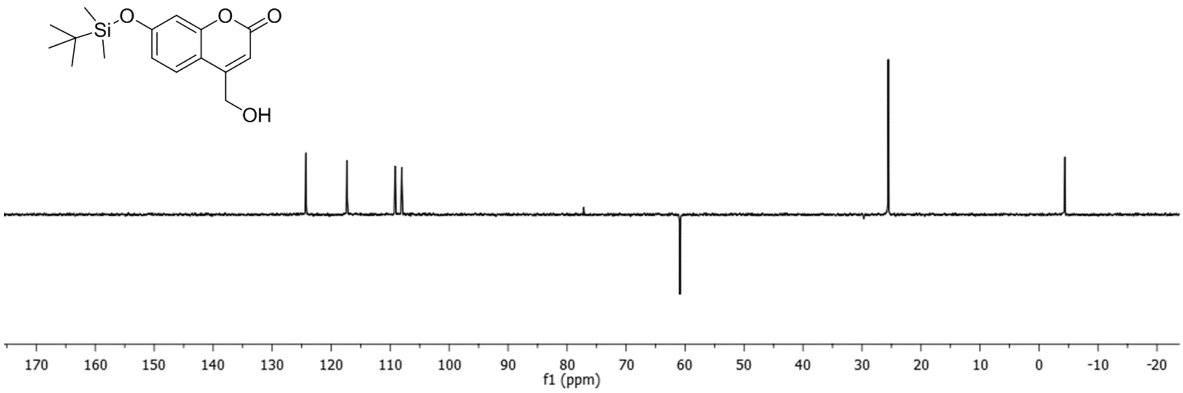

Figure S7. DEPT-135 spectrum of **4**

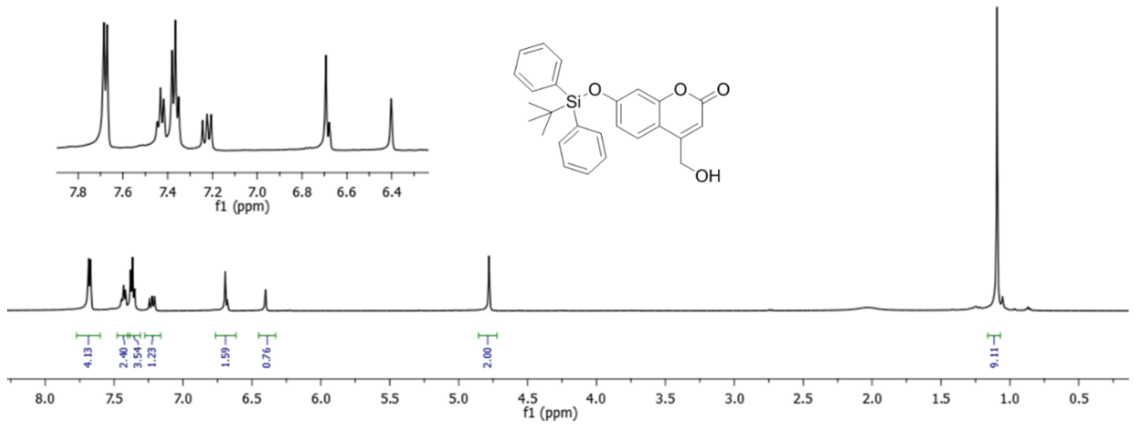

Figure S8. ^{1}H NMR spectrum of **5**

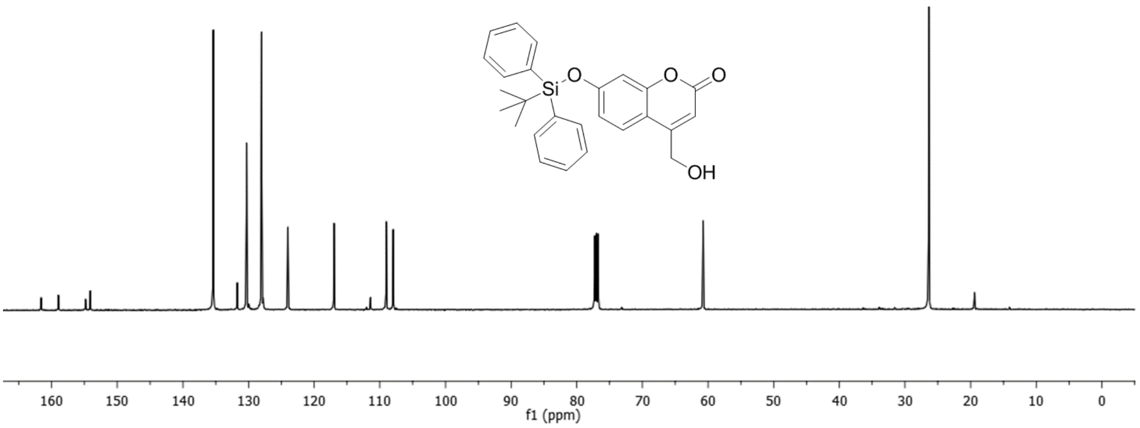

Figure S9. ^{13}C NMR spectrum of **5**

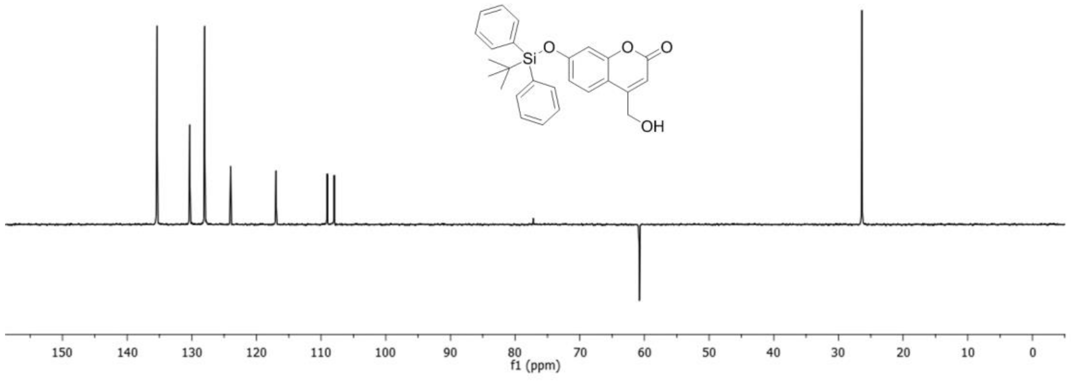

Figure S10. DEPT-135 spectrum of **5**

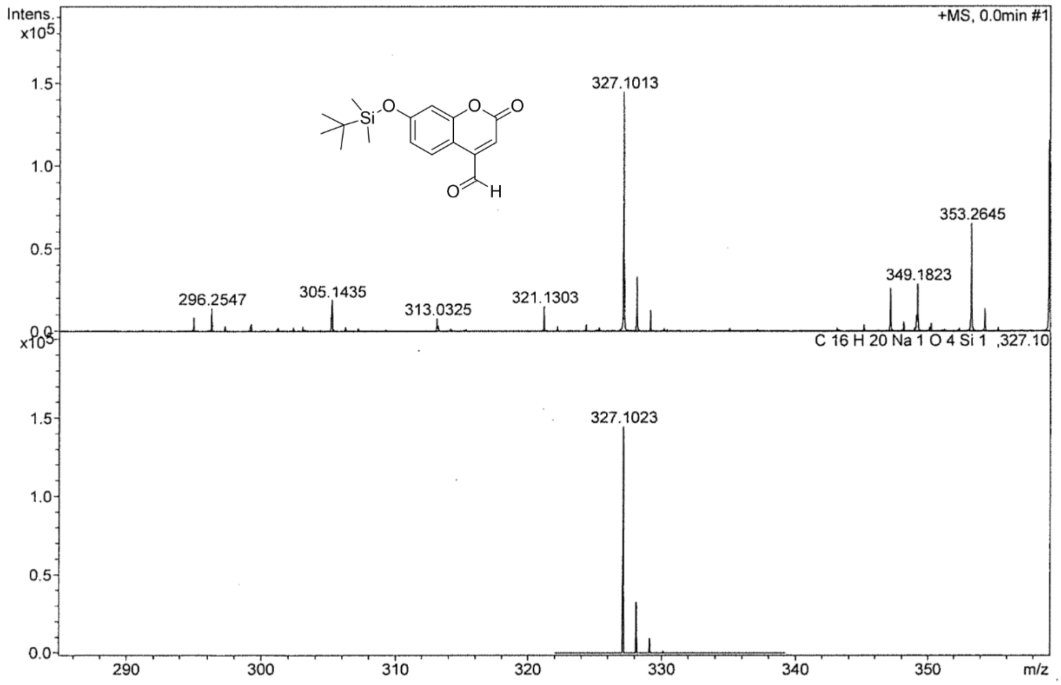

Figure S11. High resolution mass spectrum of **2**

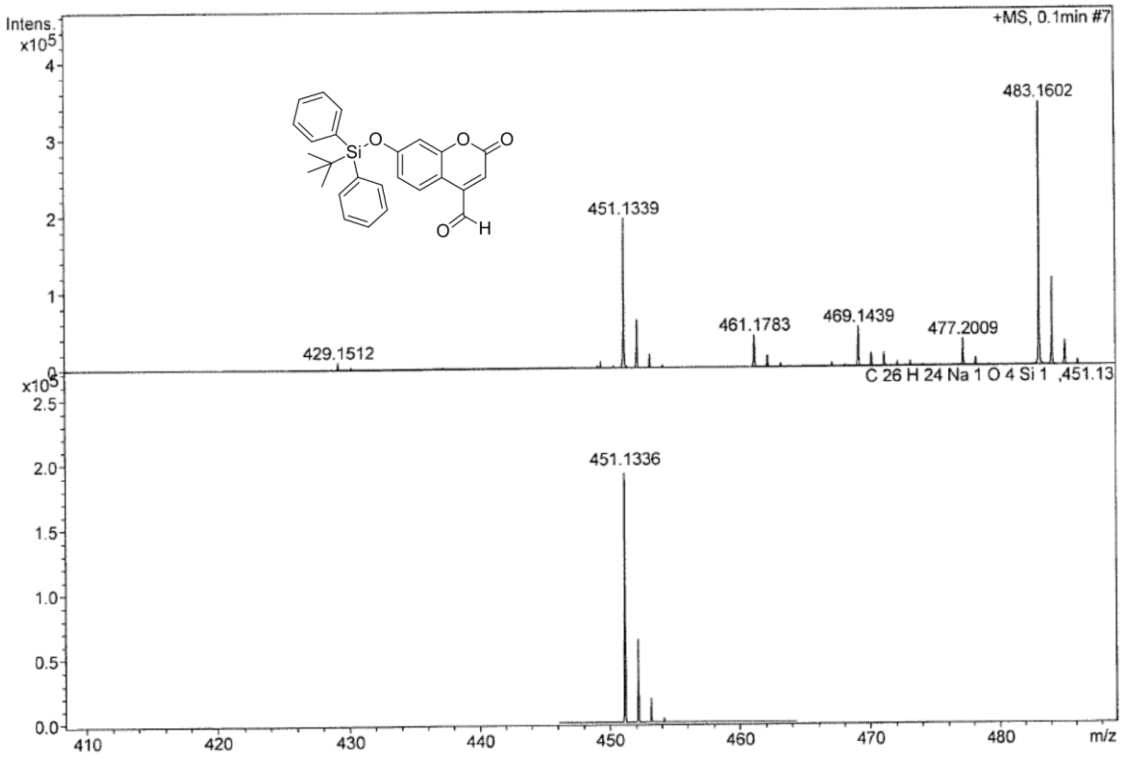

Figure S12. High resolution mass spectrum of **3**

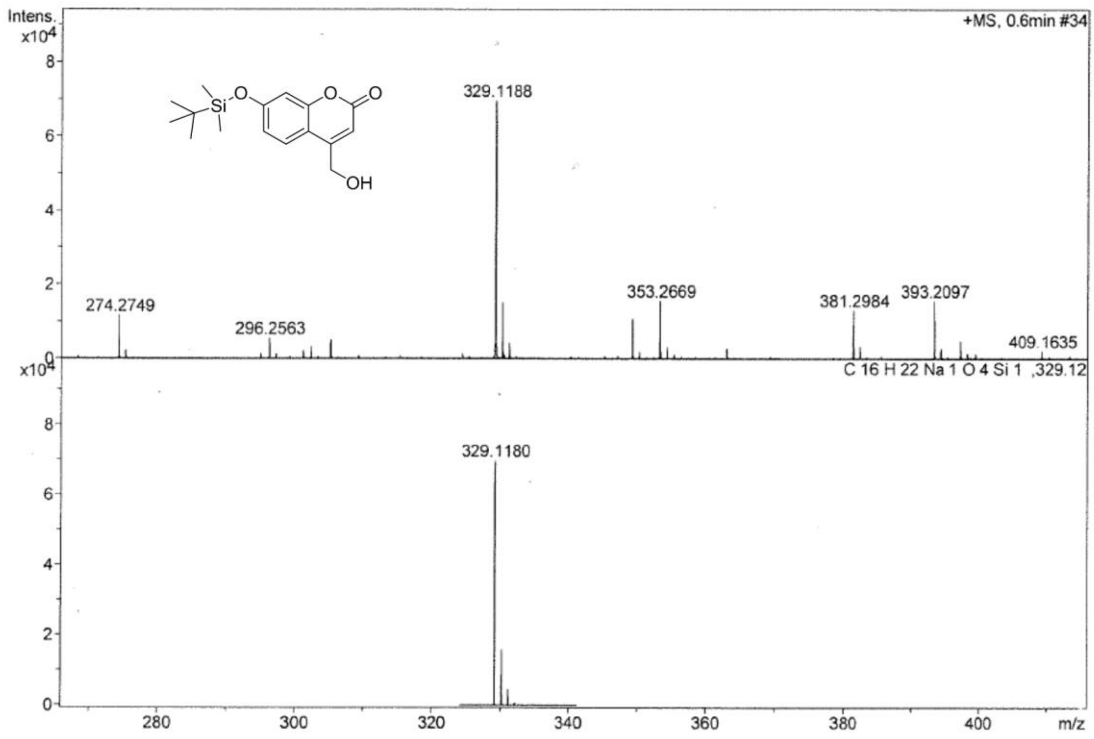

Figure S13. High resolution mass spectrum of **4**

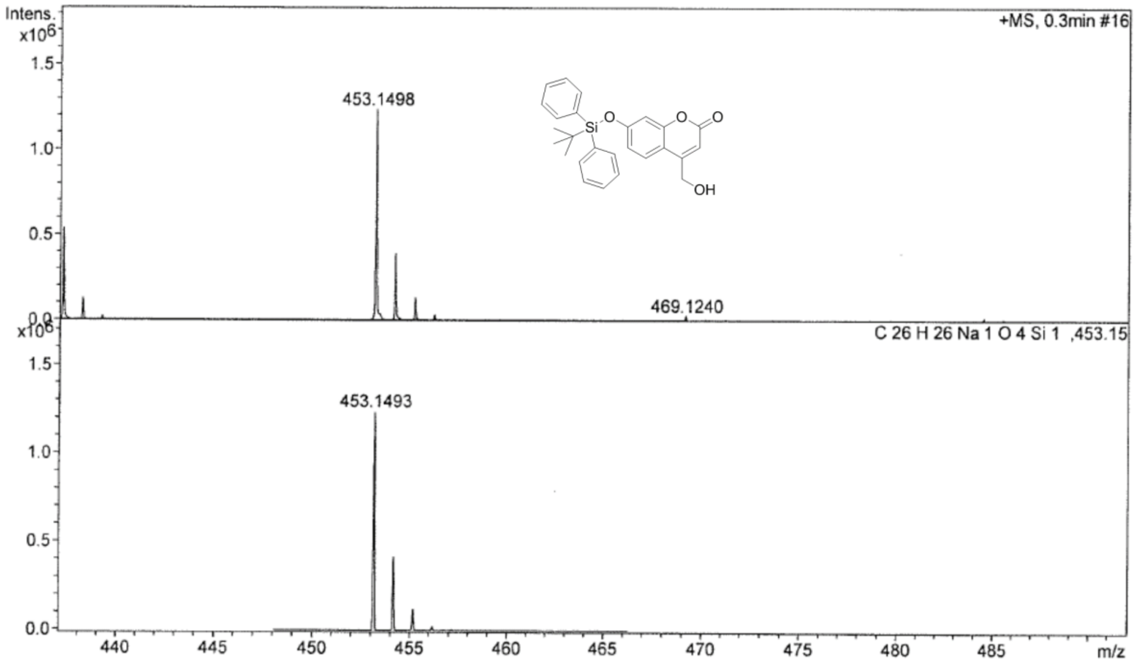

Figure S14. High resolution mass spectrum of **5**

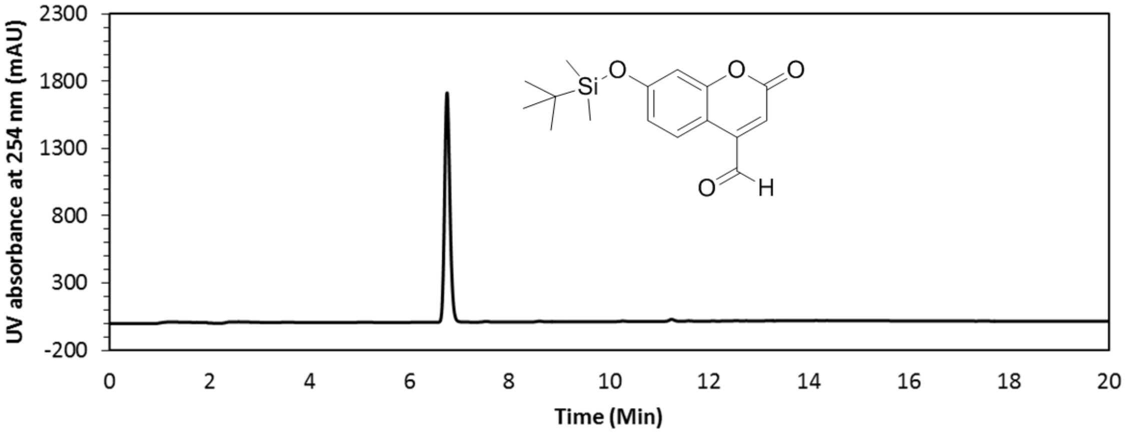

Figure S15. UV-HPLC profile of **2**

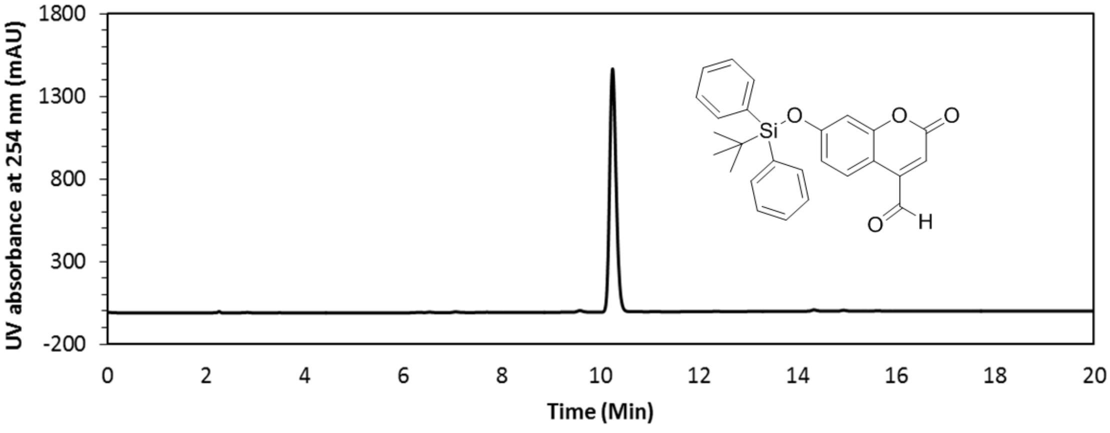

Figure S16. UV-HPLC profile of **3**

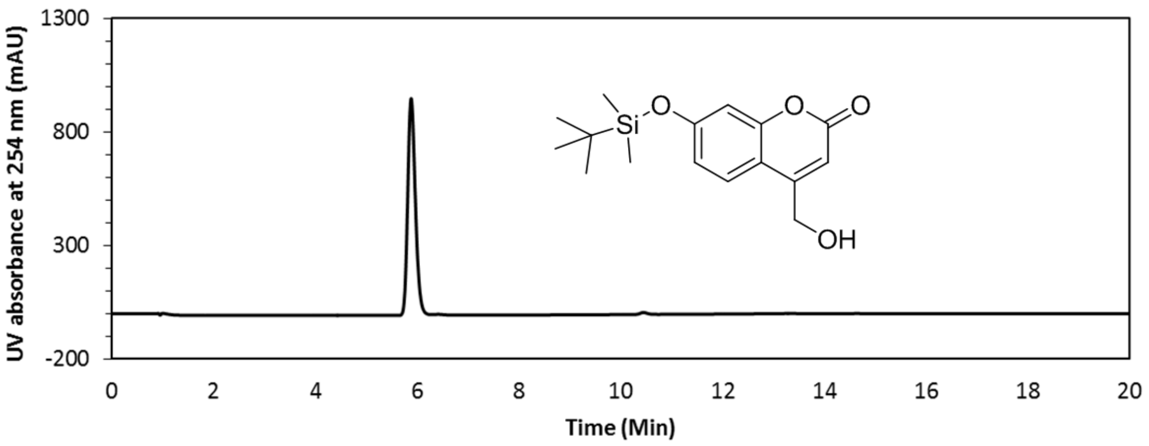

Figure S17. UV-HPLC profile of **4**

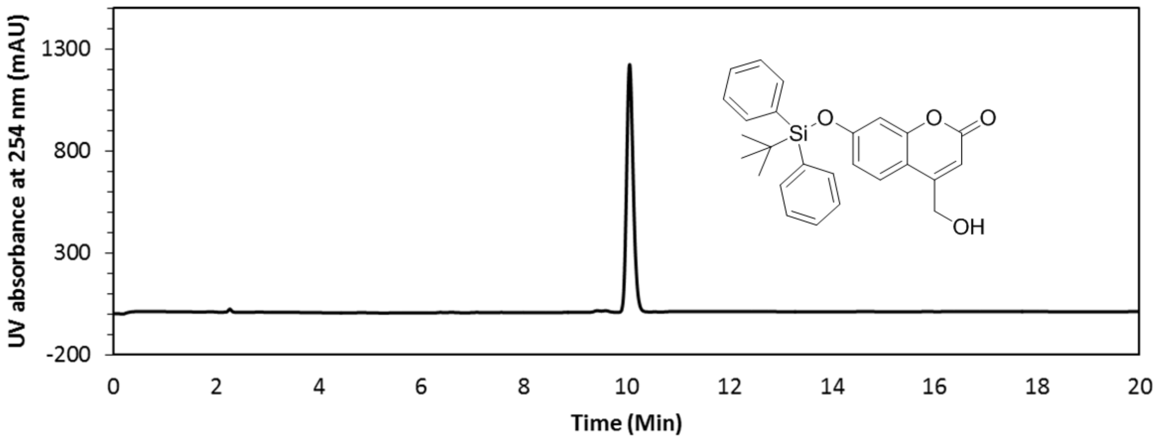

Figure S18. UV-HPLC profile of **5**

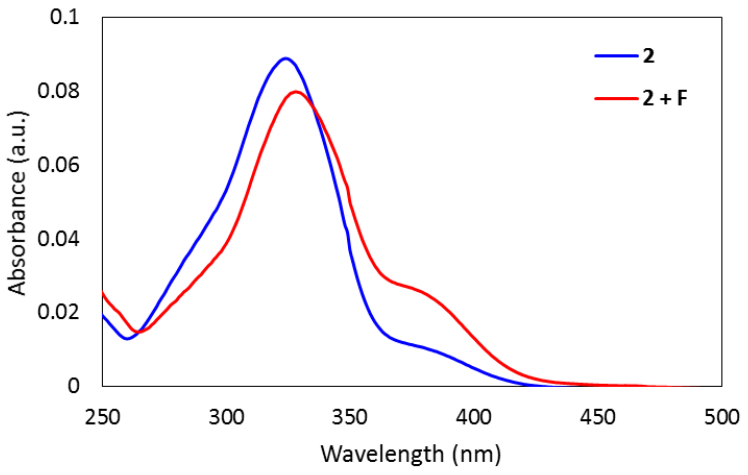

Figure S19. Absolute absorption spectra of **2** (7.2 µM) in HEPES buffer pH 7.4 (contain 3% MeCN) before (blue line) and after (red line) incubation with NaF (12.5 mM) for 1 hour

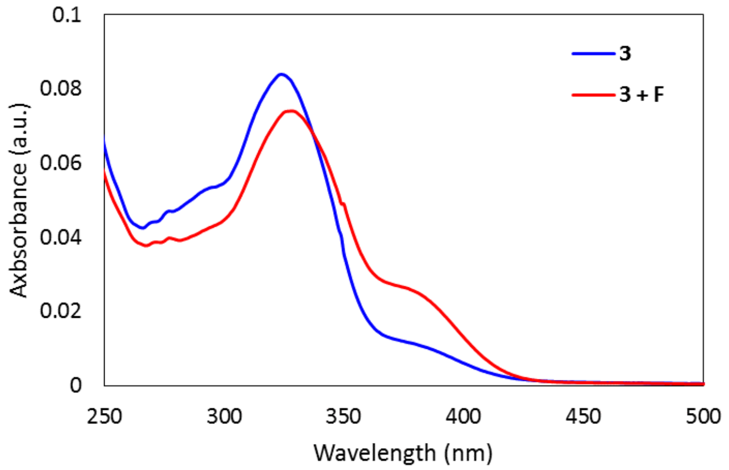

Figure S20. Absolute absorption spectra of **3** (7.2 µM) in HEPES buffer pH 7.4 (contain 3% MeCN) before (blue line) and after (red line) incubation with NaF (12.5 mM) for 1 hour

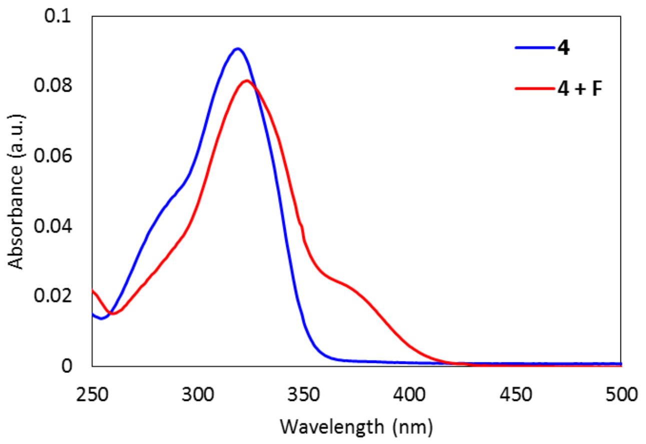

Figure S21. Absolute absorption spectra of **4** (7.2 µM) in HEPES buffer pH 7.4 (contain 3% MeCN) before (blue line) and after (red line) incubation with NaF (12.5 mM) for 1 hour

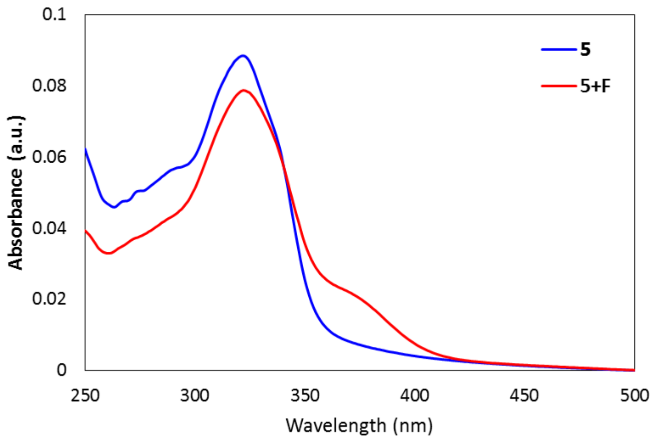

Figure S22. Absolute absorption spectra of **5** (7.2 µM) in HEPES buffer pH 7.4 (contain 3% MeCN) before (blue line) and after (red line) incubation with NaF (12.5 mM) for 1 hour

Kinetic studies of hydrolytic reactions of silyl-capped coumarins (Compound **2**, **3**, **4**, and **5**) in HEPES buffer solution pH 7.4 (contain 0.8% MeCN)

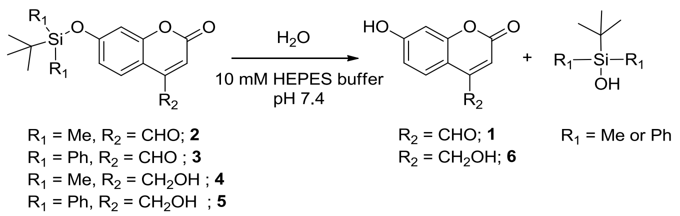

Hydrolytic reaction of silyl-capped coumarin (Coumarin_OSiR_3)

First-order rate equation: $v = k_{obs}[\text{Coumarin\_OSiR}_3]$

As the fluorescence signals increase upon desilylation, they were used to monitor the ratios of silyl-capped coumarins (Coumarin_OSiR_3) in HEPES buffer solution (contain 0.8% MeCN). All fluorescence data needed for these kinetic studies are demonstrated in **Figure S23** –**S26**

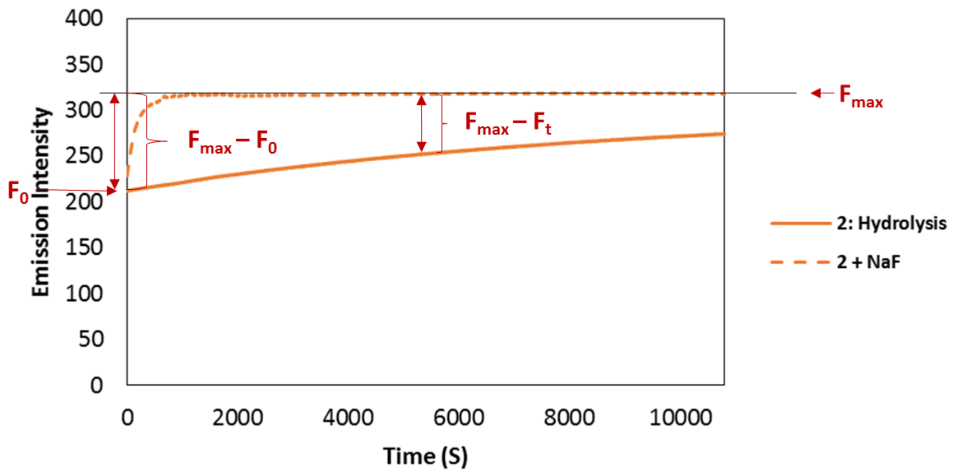

Figure S23, Fluorescence signal changes upon dissolving **2** (2 µM) in HEPES buffer solution pH 7.4 (contain 0.8% MeCN) monitored for 3 hours (Solid line) and the fluorescence signal changes upon addition of excess amount of NaF (1 mM) into the solution of **2** (2 µM) in HEPES buffer solution pH 7.4 (contain 0.8% MeCN) monitored for 3 h (dashed line).

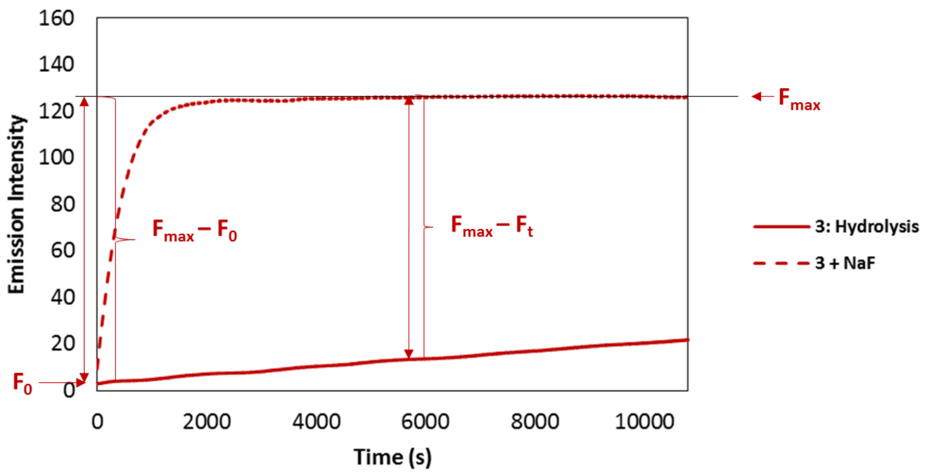

Figure S24, Fluorescence signal changes upon dissolving **3** (2 µM) in HEPES buffer solution pH 7.4 (contain 0.8% MeCN) monitored for 3 hours (Solid line) and the fluorescence signal changes upon addition of excess amount of NaF (1 mM) into the solution of **3** (2 µM) in HEPES buffer solution pH 7.4 (contain 0.8% MeCN) monitored for 3 h (dashed line).

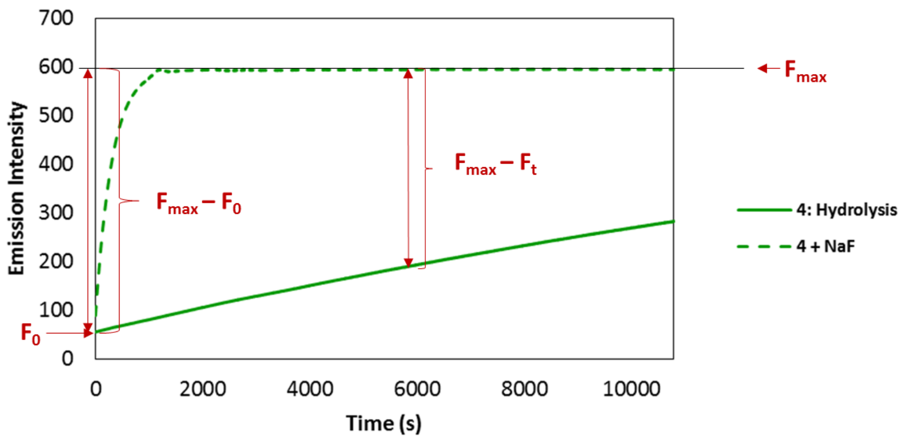

Figure S25, Fluorescence signal changes upon dissolving **4** (2 µM) in HEPES buffer solution pH 7.4 (contain 0.8% MeCN) monitored for 3 hours (Solid line) and the fluorescence signal changes upon addition of excess amount of NaF (1 mM) into the solution of **4** (2 µM) in HEPES buffer solution pH 7.4 (contain 0.8% MeCN) monitored for 3 h (dashed line).

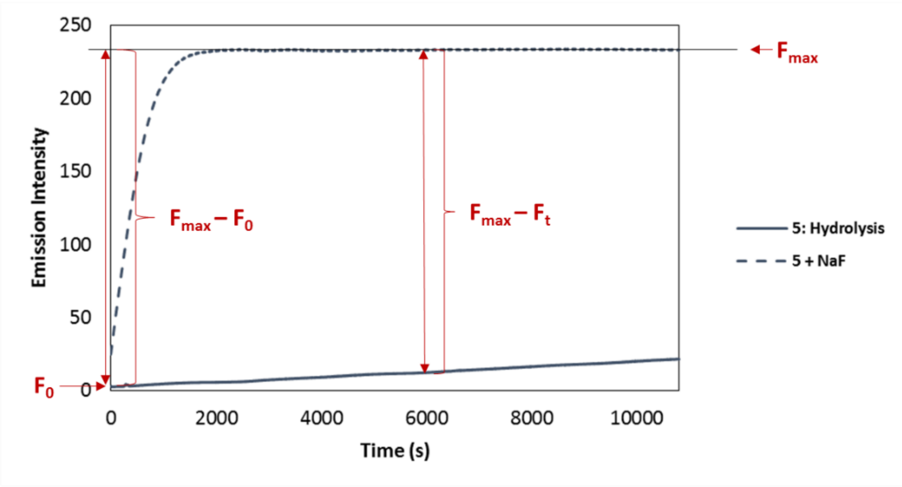

Figure S26, Fluorescence signal changes upon dissolving **5** (2 μM) in HEPES buffer solution pH 7.4 (contain 0.8% MeCN) monitored for 3 hours (Solid line) and the fluorescence signal changes upon addition of excess amount of NaF (1 mM) into the solution of **5** (2 μM) in HEPES buffer solution pH 7.4 (contain 0.8% MeCN) monitored for 3 h (dashed line).

Based on the data in Figure **S23** – **S26**;

$F_{max} – F_0$ relates to the amount of coumarin_OSiR_3 at t = 0

$F_{max} – F_t$ relates to the amount of coumarin_OSiR_3 at t = t

Where; F_{max} = Maximum fluorescence intensity after incubation of coumarin_OSiR_3 with excess amount of NaF

F_0 = Fluorescence intensity upon dissolving coumarin_OSiR_3 in HEPES buffer solution pH 7.4 (contain 0.8% MeCN) at t = 0

F_t = Fluorescence intensity upon dissolving coumarin_OSiR_3 in HEPES buffer solution pH 7.4 (contain 0.8% MeCN) at t = t

Therefore; At t = 0, Ratio of coumarin_OSiR_3 = $(F_{max} – F_0)/(F_{max} – F_0)$ = 1 = [coumarin_OSi$R_3]_0$

At t = t, Ratio of coumarin_OSiR_3 = $(F_{max} – F_t)/(F_{max} – F_0)$ = [coumarin_OSi$R_3]_t$

These data were fit to the equation [Coumarin_OSi$R_3]_t$ = [Coumarin_OSi$R_3]_0e^{-kt}$.

All kinetic data are shown in **Table S1** – **S4**.

Table S1. Kinetic data for the hydrolysis of **2**.

Compound **2**				
Time (s)	F_t (a.u.)	Exp. Ratio<br>$(F_{max} - F_t)/(F_{max} - F_0)$<br>$F_{max}$ = 317 a.u.	-ln	Calc. Ratio<br>e^(-kt)<br>k = 8.76E-5
0	212.58	1.000	0.000	1.000
500	217.16	0.956	0.045	0.957
1000	221.52	0.914	0.090	0.916
1500	226.10	0.871	0.139	0.877
2000	230.08	0.832	0.183	0.839
2500	234.29	0.792	0.233	0.803
3000	237.58	0.761	0.274	0.769
3500	241.44	0.724	0.324	0.736
4000	244.14	0.698	0.360	0.704
4500	247.39	0.667	0.405	0.674
5000	250.34	0.638	0.449	0.645
5500	253.02	0.613	0.490	0.618
6000	255.62	0.588	0.531	0.591
6500	257.93	0.566	0.570	0.566
7000	260.13	0.545	0.608	0.541
7500	262.31	0.524	0.647	0.518
8000	264.94	0.499	0.696	0.496
8500	266.67	0.482	0.730	0.475
9000	268.47	0.465	0.766	0.454
9500	270.12	0.449	0.801	0.435
10000	272.09	0.430	0.844	0.416
10500	274.08	0.411	0.889	0.398
11000				0.381
11500				0.365
12000				0.349

Table S2. Kinetic data for the hydrolysis of **3**.

Compound **3**				
Time (s)	F_t (a.u.)	Exp. Ratio $(F_{max} - F_t)/(F_{max} - F_0)$ F_{max} = 126 a.u.	-ln	Calc. Ratio e^(-kt) k = 1.56E-5
0	5.10	1.000	0.000	1.000
500	6.21	0.991	0.009	0.992
1000	6.83	0.986	0.014	0.984
1500	8.15	0.975	0.026	0.977
2000	9.14	0.967	0.034	0.969
2500	9.59	0.963	0.038	0.962
3000	10.13	0.958	0.043	0.954
3500	11.49	0.947	0.054	0.947
4000	12.50	0.939	0.063	0.939
4500	13.10	0.934	0.069	0.932
5000	14.18	0.925	0.078	0.925
5500	15.19	0.917	0.087	0.918
6000	15.64	0.913	0.091	0.910
6500	16.35	0.907	0.098	0.903
7000	17.27	0.899	0.106	0.896
7500	18.27	0.891	0.115	0.889
8000	19.10	0.884	0.123	0.882
8500	20.08	0.876	0.132	0.876
9000	20.88	0.869	0.140	0.869
9500	21.86	0.861	0.149	0.862
10000	22.37	0.857	0.154	0.855
10500	23.30	0.849	0.163	0.849
11000				0.842
11500				0.835
12000				0.829

Table S3. Kinetic data for the hydrolysis of **4**.

Compound **4**				
Time (s)	F_t (a.u.)	Exp. Ratio $(F_{max} - F_t)/(F_{max} - F_0)$ F_{max} = 630 a.u.	-ln	Calc. Ratio e^(-kt) k = 4.58E-5
0	55.69	1.000	0.000	1.000
500	68.67	0.977	0.023	0.977
1000	80.84	0.956	0.045	0.955
1500	93.34	0.934	0.068	0.934
2000	105.57	0.913	0.091	0.913
2500	117.91	0.892	0.115	0.892
3000	128.93	0.872	0.136	0.872
3500	139.81	0.854	0.158	0.852
4000	150.82	0.834	0.181	0.833
4500	161.73	0.815	0.204	0.814
5000	172.69	0.796	0.228	0.795
5500	183.18	0.778	0.251	0.777
6000	193.35	0.760	0.274	0.760
6500	203.81	0.742	0.298	0.743
7000	213.17	0.726	0.320	0.726
7500	223.18	0.708	0.345	0.709
8000	233.15	0.691	0.370	0.693
8500	242.39	0.675	0.393	0.678
9000	251.69	0.659	0.417	0.662
9500	260.46	0.643	0.441	0.647
10000	269.68	0.627	0.466	0.633
10500	277.91	0.613	0.489	0.618
11000				0.604
11500				0.591
12000				0.577

Table S4. Kinetic data for the hydrolysis of **5**.

Compound **5**				
Time (s)	F_t (a.u.)	Exp. Ratio $(F_{max} - F_t)/(F_{max} - F_0)$ F_{max} = 232 a.u.	-ln	Calc. Ratio e^(-kt) k = 7.70E-6
0	6.25	1.000	0.000	1.000
500	7.12	0.996	0.004	0.996
1000	8.31	0.991	0.009	0.992
1500	9.05	0.988	0.012	0.989
2000	9.38	0.986	0.014	0.985
2500	9.79	0.984	0.016	0.981
3000	11.03	0.979	0.021	0.977
3500	11.97	0.975	0.026	0.973
4000	12.76	0.971	0.029	0.970
4500	13.89	0.966	0.034	0.966
5000	14.91	0.962	0.039	0.962
5500	15.32	0.960	0.041	0.959
6000	16.06	0.957	0.044	0.955
6500	17.08	0.952	0.049	0.951
7000	18.12	0.947	0.054	0.948
7500	19.04	0.943	0.058	0.944
8000	20.10	0.939	0.063	0.940
8500	21.15	0.934	0.068	0.937
9000	21.85	0.931	0.072	0.933
9500	22.61	0.928	0.075	0.930
10000	23.58	0.923	0.080	0.926
10500	24.77	0.918	0.086	0.922
11000				0.919
11500				0.915
12000				0.912

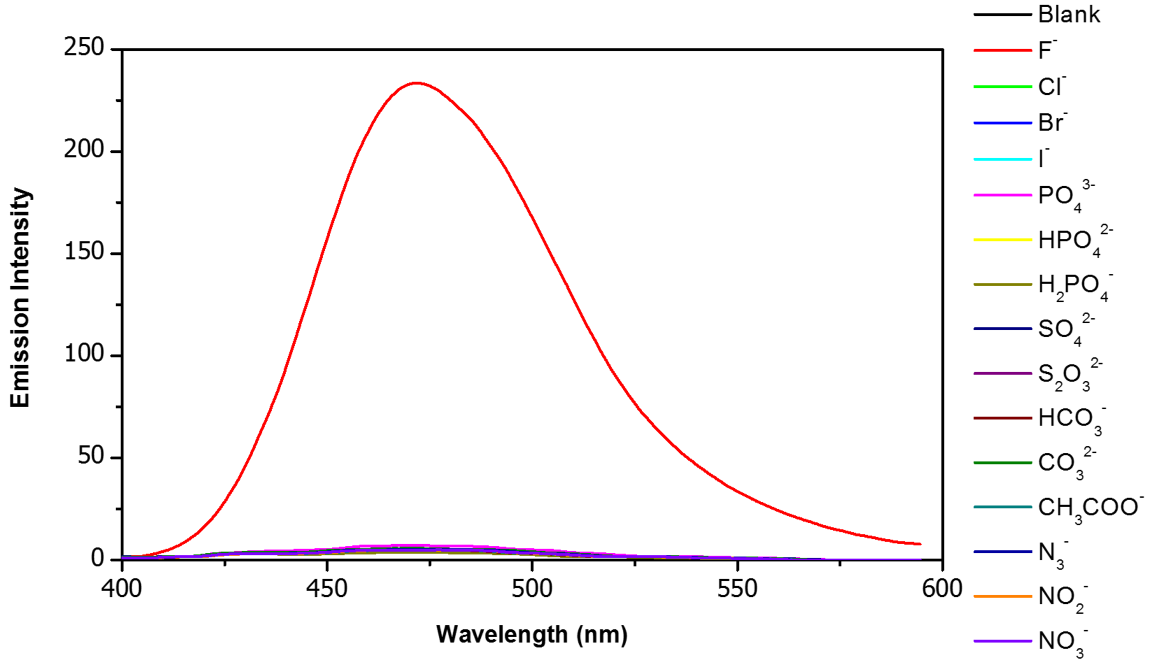

Figure S27. The fluorescence spectra of **5** (2 μM) in the presence of different anions (1 mM) in HEPES buffer solution pH 7.4 (contain 0.8% MeCN).

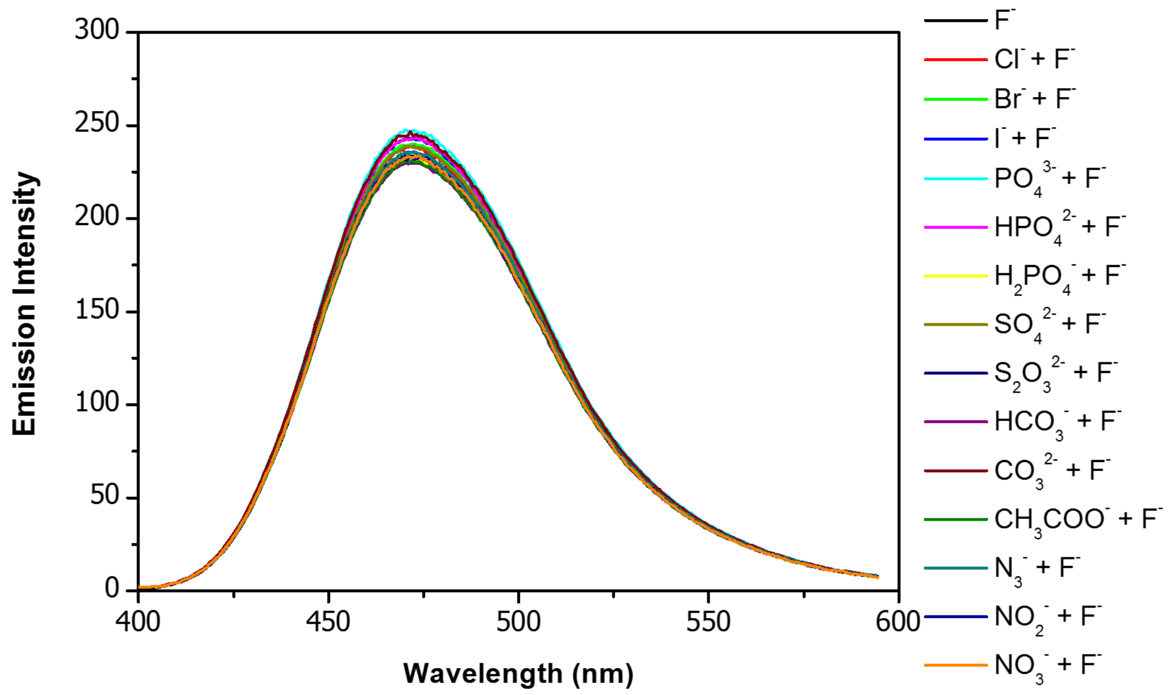

Figure S28. The fluorescence spectra of **5** (2 μM) in the presence of fluoride (1 mM) co-existing with other anions (1mM) in HEPES buffer solution pH 7.4 (contain 0.8% MeCN).

The method for determining the limit of detection:

The calibration curve was obtained from the plot of fluorescence intensity increment ($F\text{-}F_0$), as a function of the fluoride concentrations.

The limit of detection = $3 \times \sigma / m$

where *m* is the slope of the curve equation, and σ represents the standard deviation for the emission intensity of the probe solution (2 µM) in the absence of fluoride anion.

The curve equation (**Figure 8**) was determined as;

$F\text{-}F_0$ = 16.024 x [Fluoride] + 0.546 (R^2 = 0.999).

The emission intensity of the HEPES buffer solution of **5** (2 µM) in the absence of fluoride anion = 4.87 ± 0.23 (S.D.)

Therefore, the limit of detection (LOD) = (3 x 0.23)/16.024 = 0.043 ppm (43 ppb).
